# The Role of ULK3 in Cancer Progression: A Pan-Cancer Bioinformatics Analysis Integrated with Experimental Validation in Prostate Cancer

**DOI:** 10.3390/ijms27136040

**Published:** 2026-07-05

**Authors:** Yangyang Han, Mengqi Zhang, Mannizire Rehemujiang, Xintong Li, Yimin Liu, Niuniu Zhang, Meng Sun, Yunbo Zhang, Ayshamgul Hasim, Mengjia Li

**Affiliations:** 1 Department of Biology, School of Basic Medical Sciences, Xinjiang Medical University, Urumqi 830017, China; yyhan@xjmu.edu.cn (Y.H.); 13894343612@163.com (M.Z.); 18999960754@163.com (M.R.); 15098373960@163.com (X.L.); 18199840012@163.com (Y.L.); 13899065367@163.com (N.Z.); sm199901@163.com (M.S.); 18768900024@163.com (Y.Z.); 2 Xinjiang Key Laboratory of Molecular Biology for Endemic Diseases, Xinjiang Medical University, Urumqi 830017, China; axiangu@xjmu.edu.cn; 3 Key Laboratory of High Incidence Disease Research in Xingjiang, Xinjiang Medical University, Ministry of Education, Urumqi 830017, China; 4 Department of Basic Medicine, Xinjiang Medical University, Urumqi 830017, China

**Keywords:** *ULK3*, pan-cancer, prognostic biomarkers, immunity, prostate cancer

## Abstract

Unc-51-like kinase 3 (ULK3) is a key member of the ULK serine/threonine kinase family. Aberrant *ULK3* expression has been increasingly linked to tumorigenesis and malignant progression in multiple cancer types. However, the precise role of ULK3 in tumor initiation and progression remains incompletely understood. Leveraging integrated multi-omics data from The Cancer Genome Atlas (TCGA), the Genotype-Tissue Expression (GTEx) project, and the Clinical Proteomic Tumor Analysis Consortium (CPTAC), we systematically characterized the expression of ULK3 at both the transcript and protein levels across 33 cancer types. We also evaluated genomic alterations, prognostic significance, alternative splicing, pathway enrichment, tumor stemness, immune infiltration, and immunotherapy-related biomarkers. In parallel, we investigated the function of ULK3 in prostate cancer PC-3 cells using cellular localization analysis, wound-healing assays, and MTT assays. We further applied Connectivity Map (CMap) screening and molecular docking to identify candidate ULK3 activators. *ULK3* was significantly upregulated in 13 cancer types, including Bladder Urothelial Carcinoma, Breast Invasive Carcinoma, and Lung Adenocarcinoma. In contrast, *ULK3* was downregulated in Cholangiocarcinoma and Head and Neck Squamous Cell Carcinoma. High *ULK3* expression was associated with poor overall survival in Adrenocortical Carcinoma, Kidney Renal Clear Cell Carcinoma, and Skin Cutaneous Melanoma. Copy number amplification contributed to *ULK3* overexpression. A recurrent A206V missense mutation was detected in the protein kinase (Pkinase) domain. Genes co-expressed with *ULK3* were enriched in RNA splicing, methylation, oxidative phosphorylation, and energy metabolism. *ULK3* expression showed positive correlations with tumor stemness indices and m1A/m5C/m6A RNA modification regulators. From an immunological perspective, high *ULK3* expression was associated with lower Immune Score, increased M2 macrophage infiltration, and co-expression of *PD-L1*, *CTLA4*, and *LAG3* in most cancers. *ULK3* expression was also correlated with Tumor Mutational Burden in Kidney Renal Clear Cell Carcinoma and Rectum Adenocarcinoma. In addition, *ULK3* expression was associated with Microsatellite Instability in Brain Lower Grade Glioma, Lung Adenocarcinoma, and Uterine Corpus Endometrial Carcinoma. *ULK3* overexpression promoted proliferation and migration in PC-3 cells. Cephaeline was screened as a putative ULK3 activator. Overall, *ULK3* expression and amplification were associated with poor clinical outcomes, tumor stemness, immunosuppression, and RNA dysregulation. These findings highlight the potential value of *ULK3* as a pan-cancer diagnostic and prognostic biomarker and as a predictor of immunotherapy response, particularly in prostate cancer.

## 1. Introduction

At present, cancer remains one of the leading causes of death worldwide. Its marked heterogeneity, high tendency for recurrence and metastasis, and frequent drug resistance severely limit the improvement of clinical outcomes [1]. Although targeted therapy and immunotherapy have achieved major advances in certain cancers, most patients still face several critical challenges. These challenges include the lack of effective predictive markers, variable treatment responses, and inaccurate prognostic assessment. Therefore, the identification of novel biomarkers with diagnostic, prognostic, and treatment-guiding value has become a central need in cancer therapy.

The ULK (Unc-51-like kinase) family comprises a group of serine/threonine protein kinases. These kinases play important roles in autophagy initiation, cellular metabolism, and signal transduction [2]. This family includes Unc-51-like kinase 1 (ULK1), Unc-51-like kinase 2 (ULK2), and Unc-51-like kinase 3 (*ULK3*). Among them, ULK1 and ULK2 are core regulators of the canonical autophagy pathway and have been widely investigated [3]. Accumulating evidence shows that dysregulated *ULK1* expression occurs in various malignancies. *ULK1* contributes to major oncogenic processes, including tumor growth and dissemination, therapy resistance, and regulation of the tumor immune landscape. For example, *ULK1* deficiency in pancreatic ductal adenocarcinoma (PDAC) suppresses autophagic activity, inhibits tumor growth, and increases CD8+ T lymphocyte infiltration [4]. In contrast, elevated *ULK1* expression in epithelial ovarian cancer (EOC) is strongly associated with shorter overall survival [5]. In addition, ULK1 and ULK2 may negatively regulate the PTK2/SRC signaling axis by phosphorylating the focal adhesion protein PXN through a non-canonical autophagy pathway. This process suppresses focal adhesion formation, disrupts cellular mechano-transduction, and reduces the migratory capacity of breast cancer cells [6]. *ULK2* also acts as a tumor suppressor in gastric cancer. Promoter methylation-mediated silencing of *ULK2* expression may be associated with epithelial–mesenchymal transition through autophagy activation. This mechanism may promote gastric cancer cell migration and phenotypic transition toward poor differentiation [7].

The substrate specificity and regulatory network of ULK3 differ from those of ULK1 and ULK2. In cancer, *ULK3* expression and function appear to be tissue-specific. In squamous cell carcinoma (SCC), *ULK3* is upregulated. *ULK3* silencing significantly inhibits tumor cell proliferation, colony formation, and in vivo tumorigenicity. The underlying mechanism involves regulation of PRMT1/5 activity, which alters histone methylation and modulates stem cell-associated signaling pathways [8]. In breast cancer, *ULK3* expression shows greater complexity. Its overall expression level is lower in tumor tissues than in matched normal tissues. In vitro studies have shown that *ULK3* overexpression suppresses the proliferation and migration of MCF-7 cells [9]. Moreover, *ULK3* upregulation activates autophagy and induces neuronal injury in an Alzheimer’s disease model. In contrast, *ULK3* inhibition effectively alleviates the related pathological phenotypes [10]. These findings indirectly suggest that ULK3 is involved in cellular homeostasis. Current evidence indicates that ULK3 may function as either a tumor-promoting or tumor-suppressive factor in different cancer types. This dual role highlights its potential importance as a prognostic biomarker and therapeutic target.

Although *ULK1* and *ULK2* have been extensively studied in multiple cancers and have shown clear clinical potential, *ULK3* remains largely unexplored in the pan-cancer context. Existing studies are mainly limited to individual cancer types, such as SCC and breast invasive carcinoma (BRCA). Comprehensive analyses of *ULK3* expression profiles, prognostic associations, and immune-regulatory mechanisms across cancers remain insufficient. Large-scale multi-omics databases, including The Cancer Genome Atlas Program (TCGA), Genotype-Tissue Expression (GTEx), and the Clinical Proteomic Tumor Analysis Consortium (CPTAC), provide valuable resources for such analyses. ULK3 may exert opposite functional effects in different tumor types, such as tumor-promoting effects in SCC and tumor-suppressive effects in BRCA [8,9]. Moreover, the role of ULK3 in shaping the tumor immune microenvironment remains poorly understood. Its potential influence on immune checkpoint expression, immunotherapy response, and downstream signaling networks also requires further investigation. Therefore, this study comprehensively evaluates *ULK3* expression patterns, prognostic significance, and immune-regulatory mechanisms across multiple cancer types. This study aims to provide a theoretical basis and empirical evidence for the potential clinical application of ULK3 as a functional biomarker in oncology.

The role of *ULK3* in prostate cancer remains unclear. Our group has long focused on basic and translational research in prostate cancer. In previous studies, we showed that prostate cancer cell lines are suitable models for validating pan-cancer oncogene functions. In the present study, we analyzed *ULK3* expression across multiple cancer types. We found that *ULK3* was significantly upregulated in prostate cancer. High *ULK3* expression was significantly associated with poor progression-free survival in patients with prostate cancer. *ULK3* expression was also closely related to multiple immune checkpoint molecules and indicators of an immunosuppressive microenvironment. Therefore, we further investigated the expression characteristics, clinical significance, and potential biological functions of ULK3 in prostate cancer cells in vitro. These analyses aimed to provide experimental evidence supporting ULK3 as a potential biomarker and therapeutic target in prostate cancer. This rationale explains why prostate cancer was selected as the validation model.

## 2. Results

### 2.1. Differential Expression Analysis of ULK3 in Pan-Cancer

**Figure 1:**
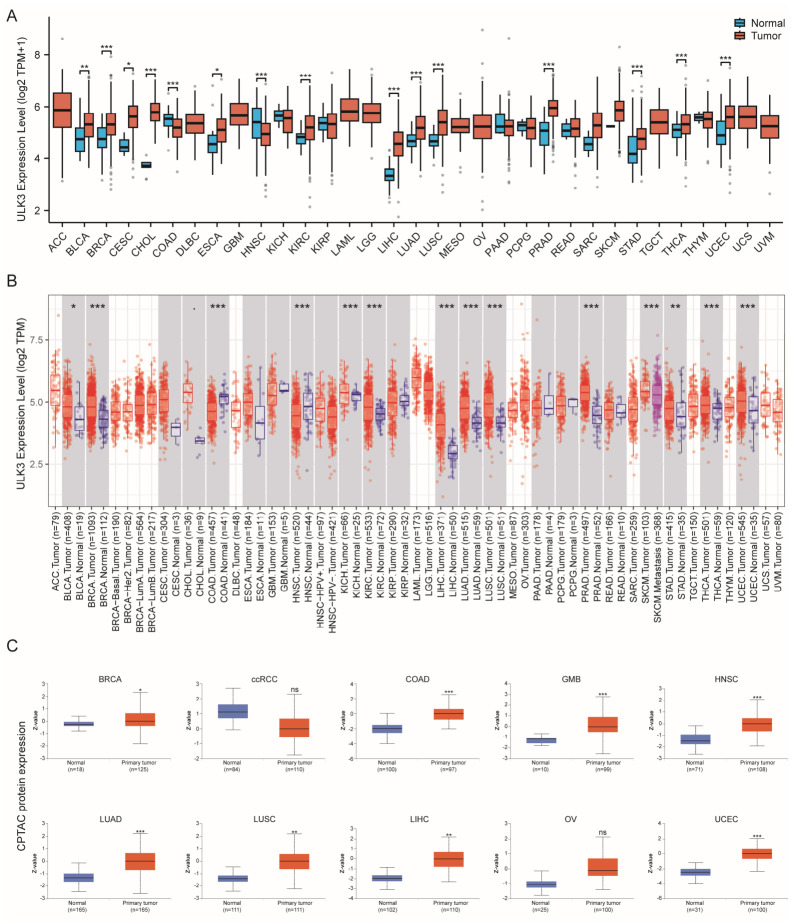
Differential expression of *ULK3*. (**A**) This panel shows the comparative analysis of *ULK3* mRNA expression between TCGA cancer tissues and GTEx normal tissues. Red bars represent cancer samples, and blue bars represent normal samples. The normal group includes normal tissues from the TCGA and GTEx databases. Differential expression analysis was performed using the edgeR package and Welch’s *t*-test, with multiple comparisons adjusted by false discovery rate (FDR) correction. (**B**) This panel shows *ULK3* mRNA expression across different cancer types based on TIMER. The edgeR package was used, and the results are presented as log2 TPM. FDR correction was applied for multiple comparisons. (**C**) This panel shows the comparative analysis of ULK3 protein expression between normal tissues and primary tumor tissues across 10 cancer types using A Portal for Facilitating Tumor Subgroup Gene Expression and Survival Analyses (UALCAN). Welch’s *t*-test was used, and FDR correction was applied. * *p* < 0.05, ** *p* < 0.01, *** *p* < 0.001. The normal groups were defined as normal tissues from the TCGA database.

**Figure 2:**
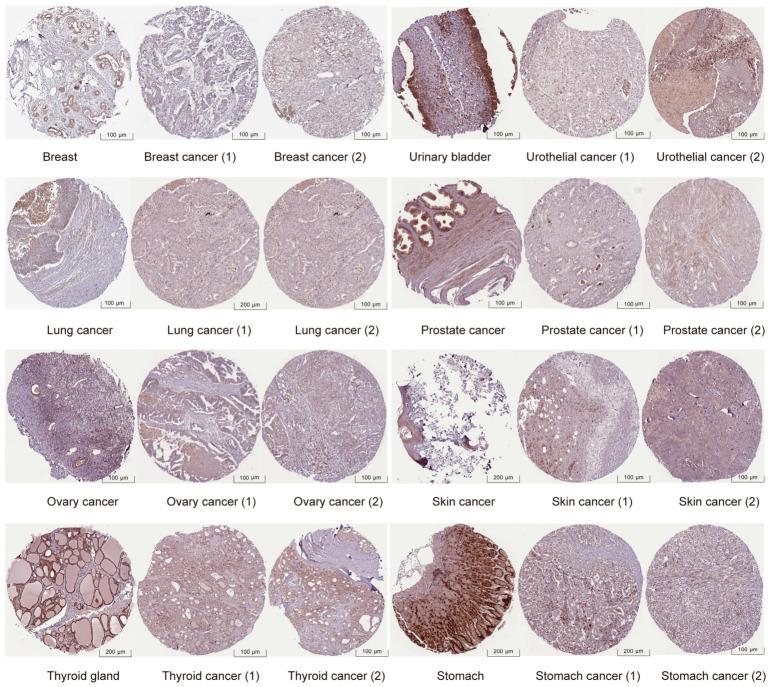
Protein expression of ULK3 in immunohistochemical images from normal tissues and tumor tissues. The (**left image**) in each group shows normal tissue, and the (**right image**) shows tumor tissue. Each panel represents ULK3 protein expression in a different tissue type, including breast cancer, urothelial cancer, lung cancer, prostate cancer, ovarian cancer, skin cancer, thyroid cancer, and stomach cancer.

We investigated the mRNA expression levels of *ULK3* in normal human tissues and tumor tissues by analyzing RNA sequencing (RNA-seq) data from the TCGA and GTEx databases. Our analysis revealed marked heterogeneity in *ULK3* expression across 33 cancer types, after excluding malignancies without matched normal tissue controls. Compared with normal controls, *ULK3* expression was significantly increased in Bladder Urothelial Carcinoma (BLCA, *p* = 0.0016), Breast Invasive Carcinoma (BRCA, *p* = 2.01 × 10^−19^), Cervical Squamous Cell Carcinoma and Endocervical Adenocarcinoma (CESC, *p* = 0.0126), Colon Adenocarcinoma (COAD, *p* = 1.34 × 10^−5^), Esophageal Carcinoma (ESCA, *p* = 0.041), Kidney Renal Clear Cell Carcinoma (KIRC, *p* = 5.58 × 10^−8^), Liver Hepatocellular Carcinoma (LIHC, *p* = 2.71 × 10^−23^), Lung Adenocarcinoma (LUAD, *p* = 2.58 × 10^−11^), Lung Squamous Cell Carcinoma (LUSC, *p* = 2.35 × 10^−13^), Prostate Adenocarcinoma (PRAD, *p* = 3.20 × 10^−21^), Stomach Adenocarcinoma (STAD, *p* = 0.001), Thyroid Carcinoma (THCA, *p* = 0.0001), and Uterine Corpus Endometrial Carcinoma (UCEC, *p* = 0.0001) (Figure 1A). In contrast, *ULK3* expression was significantly decreased in Cholangiocarcinoma (CHOL, *p* < 0.001) and Head and Neck Squamous Cell Carcinoma (HNSC, *p* < 0.001) compared with the corresponding normal tissues (Figure 1A, Supplementary Table S2). To further assess the pan-cancer expression pattern of *ULK3*, we analyzed *ULK3* mRNA expression across different tumor types using the Tumor Immune Estimation Resource (TIMER) database. TIMER analysis showed that *ULK3* expression was significantly higher in Bladder Urothelial Carcinoma (BLCA, *p* < 0.05), Breast Invasive Carcinoma (BRCA, *p* < 0.001), Colon Adenocarcinoma (COAD, *p* < 0.001), Kidney Chromophobe (KICH, *p* < 0.001), Kidney Renal Clear Cell Carcinoma (KIRC, *p* < 0.001), Liver Hepatocellular Carcinoma (LIHC, *p* < 0.001), Lung Adenocarcinoma (LUAD, *p* < 0.001), Lung Squamous Cell Carcinoma (LUSC, *p* < 0.001), Prostate Adenocarcinoma (PRAD, *p* < 0.001), Skin Cutaneous Melanoma (SKCM, *p* < 0.001), Stomach Adenocarcinoma (STAD, *p* < 0.01), Thyroid Carcinoma (THCA, *p* < 0.001), and Uterine Corpus Endometrial Carcinoma (UCEC, *p* < 0.001) (Figure 1B, Supplementary Table S2). We also compared ULK3 protein expression between primary tumor tissues and normal tissues using the UALCAN database. This database includes proteomic data across multiple cancer types. The results showed that ULK3 protein expression was significantly higher in eight cancer types, including Breast Invasive Carcinoma (BRCA, *p* = 3.14 × 10^−2^), Colon Adenocarcinoma (COAD, *p* = 3.45 × 10^−5^), Glioblastoma Multiforme (GBM, *p* = 2.94 × 10^−15^), Head and Neck Squamous Cell Carcinoma (HNSC, *p* = 2.19 × 10^−26^), Lung Adenocarcinoma (LUAD, *p* = 2.79 × 10^−7^), Lung Squamous Cell Carcinoma (LUSC, *p* = 1.55 × 10^−3^), Liver Hepatocellular Carcinoma (LIHC, *p* = 1.04 × 10^−3^), and Uterine Corpus Endometrial Carcinoma (UCEC, *p* = 1.22 × 10^−6^) (Figure 1C, Supplementary Table S3). We further examined ULK3 protein expression using immunohistochemical staining images from The Human Protein Atlas (HPA) public database. ULK3 protein showed differential expression in eight cancer types compared with the corresponding normal tissues (Figure 2). These immunohistochemical findings were generally consistent with the mRNA expression results shown in Figure 1. Collectively, these data indicate that *ULK3* expression is significantly altered in multiple cancer types. The increased expression of *ULK3* in many tumors suggests that *ULK3* may be involved in tumorigenesis. These findings also support its potential value as a candidate diagnostic biomarker in specific cancer types. Furthermore, these observations highlight the need for a comprehensive pan-cancer analysis to clarify the broader oncological significance of ULK3.

### 2.2. Analysis of the Correlation Between ULK3 Expression and Pan-Cancer Prognosis

**Figure 3:**
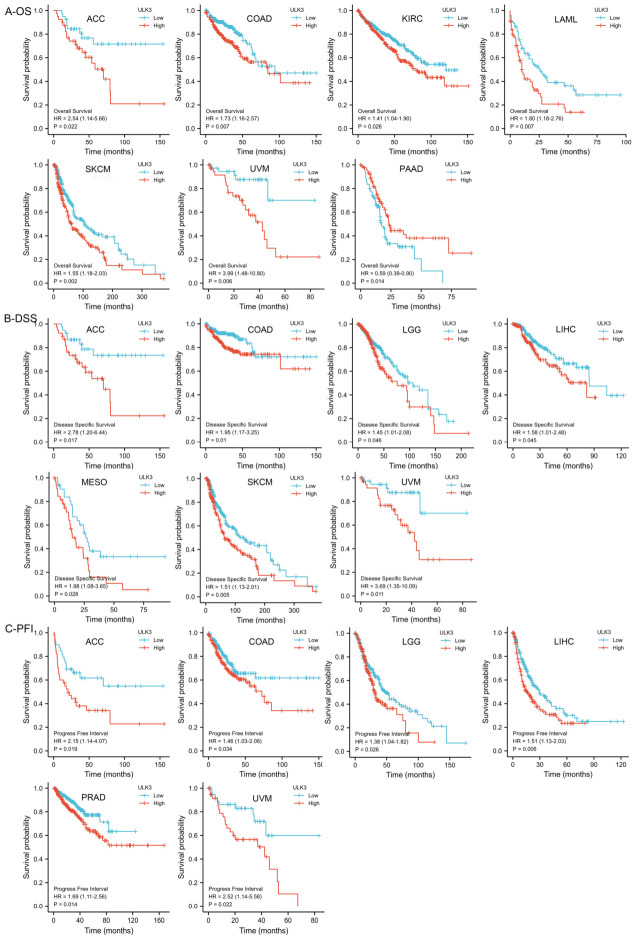
Overall survival (OS), disease-specific survival (DSS), and progression-free interval (PFI) predicted by Kaplan–Meier curves in TCGA patients. (**A**) This panel shows the relationship between *ULK3* expression and OS. (**B**) This panel shows the relationship between *ULK3* expression and DSS. (**C**) This panel shows the relationship between *ULK3* expression and PFI in TCGA patients. Kaplan–Meier survival analysis was performed using the log-rank test. The R packages survival v3.3.1 and survminer v0.4.9 were used.

We further analyzed the association between *ULK3* expression and patient prognosis across multiple cancer types. In the overall survival (OS) analysis, high *ULK3* expression was significantly associated with shorter OS in ACC (HR = 2.54, 95% CI: 1.14–5.66, *p* = 0.0221), KIRC (HR = 1.14, 95% CI: 1.04–1.90, *p* = 0.0291), LAML (HR = 1.80, 95% CI: 1.18–2.76, *p* = 0.0072), SKCM (HR = 1.55, 95% CI: 1.18–2.03, *p* = 0.0021), and UVM (HR = 3.99, 95% CI: 1.48–10.80, *p* = 0.0064). In contrast, *ULK3* expression was negatively associated with OS in PAAD (HR = 0.59, 95% CI: 0.39–0.90, *p* = 0.0031) (Figure 3A and Supplementary Figure S1A). In the disease-specific survival (DSS) analysis, high *ULK3* expression was significantly associated with poorer outcomes in ACC (HR = 2.78, 95% CI: 1.20–6.44, *p* = 0.0170), LGG (HR = 1.45, 95% CI: 1.01–2.08, *p* = 0.0413), MESO (HR = 1.98, 95% CI: 1.08–3.65, *p* = 0.0308), SKCM (HR = 1.51, 95% CI: 1.13–2.01, *p* = 0.0074), and UVM (HR = 3.69, 95% CI: 1.35–10.09, *p* = 0.0108) (Figure 3B and Supplementary Figure S1B). Moreover, elevated *ULK3* expression was correlated with a shorter progression-free interval (PFI) in ACC (HR = 2.15, 95% CI: 1.14–4.07, *p* = 0.0186), LGG (HR = 1.38, 95% CI: 1.04–1.82, *p* = 0.0399), LIHC (HR = 1.51, 95% CI: 1.13–2.03, *p* = 0.0061), PRAD (HR = 1.69, 95% CI: 1.11–2.56, *p* = 0.0275), UVM (HR = 2.52, 95% CI: 1.14–5.58, *p* = 0.0219) (Figure 3C, Supplementary Table S4 and Supplementary Figure S1C). To determine whether *ULK3* expression independently influenced patient prognosis, we performed multivariate Cox regression analyses. We adjusted for clinicopathological covariates, including age, tumor stage, pathological grade, and sex. The results showed that *ULK3* expression remained an independent prognostic factor for OS in ACC (HR = 2.14, 95% CI: 1.23–3.72, *p* = 0.007), KIRC (HR = 1.68, 95% CI: 1.12–2.51, *p* = 0.012), and SKCM (HR = 1.89, 95% CI: 1.18–3.02, *p* = 0.008). These associations were independent of conventional clinicopathological parameters. Taken together, these results demonstrate a strong correlation between *ULK3* expression and poor clinical prognosis in diverse malignancies.

We also conducted a systematic analysis to examine whether *ULK3* mRNA expression was associated with key clinicopathological parameters across multiple tumor types. In ACC, STAD, and SKCM, tumor stage was significantly associated with *ULK3* expression levels. Notably, *ULK3* expression was significantly associated with pathological stage in BLCA, SKCM, and TGCT (Supplementary Figure S2). Kaplan–Meier survival analysis further revealed that *ULK3* expression was associated with prognosis in multiple cancer types, including BLCA (HR = 0.67, 95% CI: 0.49–0.92, *p* = 0.012), EAC (HR = 0.34, 95% CI: 0.15–0.77, *p* = 0.0074), ESCC (HR = 0.41, 95% CI: 0.18–0.95, *p* = 0.031), HNSC (HR = 0.66, 95% CI: 0.51–0.86, *p* = 0.0022), KIRC (HR = 1.84, 95% CI: 1.35–2.50, *p* = 1 × 10^−4^), KIRP (HR = 0.49, 95% CI: 0.27–0.88, *p* = 0.015), OV (HR = 0.76, 95% CI: 0.59–0.99, *p* = 0.042), PDAC (HR = 0.48, 95% CI: 0.31–0.73, *p* = 5 × 10^−4^), SARC (HR = 0.64, 95% CI: 0.43–0.96, *p* = 0.028), SCC (HR = 0.54, 95% CI: 0.34–0.87, *p* = 0.01), STAD (HR = 0.65, 95% CI: 0.47–0.90, *p* = 0.0097), and THYM (HR = 5.9, 95% CI: 1.47–23.63, *p* = 0.0044). Patients with reduced *ULK3* expression showed markedly worse overall survival outcomes in the corresponding survival analyses (Supplementary Figure S3). These findings suggest that ULK3 may function as a tumor suppressor in specific cancer contexts and may influence both cancer aggressiveness and clinical prognosis.

### 2.3. Genomic Variation and Epigenetic Characteristics of ULK3

**Figure 4:**
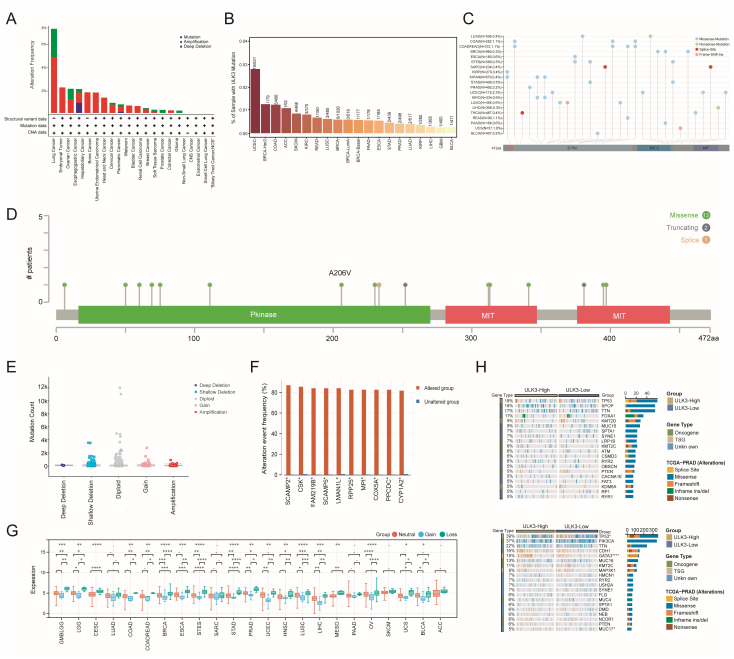
Distinct genomic profile analysis of *ULK3* across different cancers. (**A**) This panel shows the alteration frequency of *ULK3* in different cancer types. The data were obtained from the cBio Cancer Genomics Portal (cBioPortal) database and show the percentages of samples with *ULK3* mutations, structural alterations, amplifications, and deep deletions across different cancer types. (**B**) This panel shows the mutation frequency of *ULK3* in different cancer types. Data from the Tumor Immune Estimation Resource 2.0 (TIMER2.0) show the percentage of *ULK3* mutations across various cancers. (**C**) This panel shows the mutation types and distribution of *ULK3* across different cancers. The data were analyzed using the SangerBox portal and show the percentages of missense mutations, nonsense mutations, and splice-site mutations in different cancer types. (**D**) This panel shows the protein domain analysis of *ULK3* mutations. The mutation map generated by cBioPortal shows the distribution of mutation types in the Mito-carr domain and the T127M site. (**E**) This panel shows the types of *ULK3* alterations detected in pan-cancer. (**F**) This panel shows the alteration frequency of relevant genes in the *ULK3*-altered and unaltered groups. Fisher’s exact test was used. (**G**) This panel shows the analysis of *ULK3* mRNA expression changes in different cancer types. The results were obtained from SangerBox, which compared neutral, increased, and decreased copy number changes across various cancers. Pearson correlation analysis was performed with FDR correction. (**H**) This panel shows the relationship between *ULK3* expression and somatic mutations in PRAD (**top**) and BRCA (**bottom**). Data from the Comprehensive Analysis on Multi-Omics of Immunotherapy in Pan-Cancer (CAMOIP) show the percentages of oncogenes, tumor suppressor genes, and genes with unknown functions. The data also show splice-site, missense, frameshift, and nonsense mutations in the *ULK3*-high and *ULK3*-low groups. Fisher’s exact test was performed with FDR correction. * *p* < 0.05, ** *p* < 0.01, *** *p* < 0.001, **** *p* < 0.0001.

We next analyzed the genomic variation and epigenetic characteristics of *ULK3* across cancers. Pan-cancer analysis based on SangerBox and TIMER 2.0 showed that *ULK3* had a significant mutation frequency (>6%) and structural variant frequency (>4%) in lung cancer. Significant structural variant frequencies (>2%) were also detected in embryonal tumors, ovarian cancer, and esophagogastric cancer (Figure 4A). Among the cancer samples, UCEC showed the highest proportion of *ULK3* mutations (2.825%), followed by BRCA (1.266%), COAD (1.232%), ACC (1.086%), SKCM (0.855%), and KIRC (0.811%). Missense mutations were the predominant mutation type (Figure 4B). Further analysis using cBioPortal and SangerBox showed that both missense mutations and deep deletions of *ULK3* were detectable. Gene amplification was the most frequent genomic alteration. Missense mutations mainly occurred in the protein kinase (Pkinase) domain, and A206V represented the predominant variant. Collectively, these results indicate the potential utility of *ULK3* genomic alterations as candidate biomarkers for clinical diagnosis (Figure 4C–E, Supplementary Table S5).

The most common copy number variation patterns were copy number gain and diploid status. Subsequent analysis using SangerBox showed that higher *ULK3* copy number was significantly correlated with increased *ULK3* mRNA expression in 18 different cancer types, including GBMLGG, LGG, CESC, COAD, COADREAD, BRCA, ESCA, STAD, PRAD, UCEC, HNSC, LIHC, LUSC, MESO, UCS, OV, and BLCA (Figure 4G, Supplementary Table S5). These findings indicate that copy number alterations may represent a major genomic mechanism regulating *ULK3* expression.

Against the background of *ULK3* genomic alterations, co-occurrence gene analysis showed that the mutation frequencies of *SCAMP2*, *CSK*, *FAM219B*, *SCAMP5*, *LMAN1L*, *RPP25*, *MPI*, *COX5A*, *PPCDC*, and *CYP1A2* were significantly increased in the *ULK3*-altered group. These observations suggest that these molecules may cooperate with ULK3 in oncogenic signaling or jointly modulate malignant progression (Figure 4F). Finally, we used the cBioPortal web resource to investigate potential correlations between *ULK3* expression levels and somatic mutation profiles. Across a broad spectrum of malignancies, *ULK3* expression showed a statistically significant association with tumor mutational burden. In BRCA, the high-*ULK3*-expression group exhibited elevated frequencies of somatic mutations in *TP53* (39%), *GATA3* (15%), and *MUC17* (5%) (Figure 4H, bottom).

In conclusion, genomic alterations in *ULK3*, including point mutations, copy number gains, and copy number losses, were observed across diverse tumor types. Missense mutations and copy number alterations represented the predominant forms of single-nucleotide variants and large-scale genomic structural alterations, respectively. These genomic changes may be involved in key oncogenic processes, including tumor initiation, growth, and progression. They may also serve as useful indicators for cancer diagnosis and prognostic assessment.

### 2.4. The Role of ULK3 Alternative Splicing in Determining Cancer Outcomes

**Figure 5:**
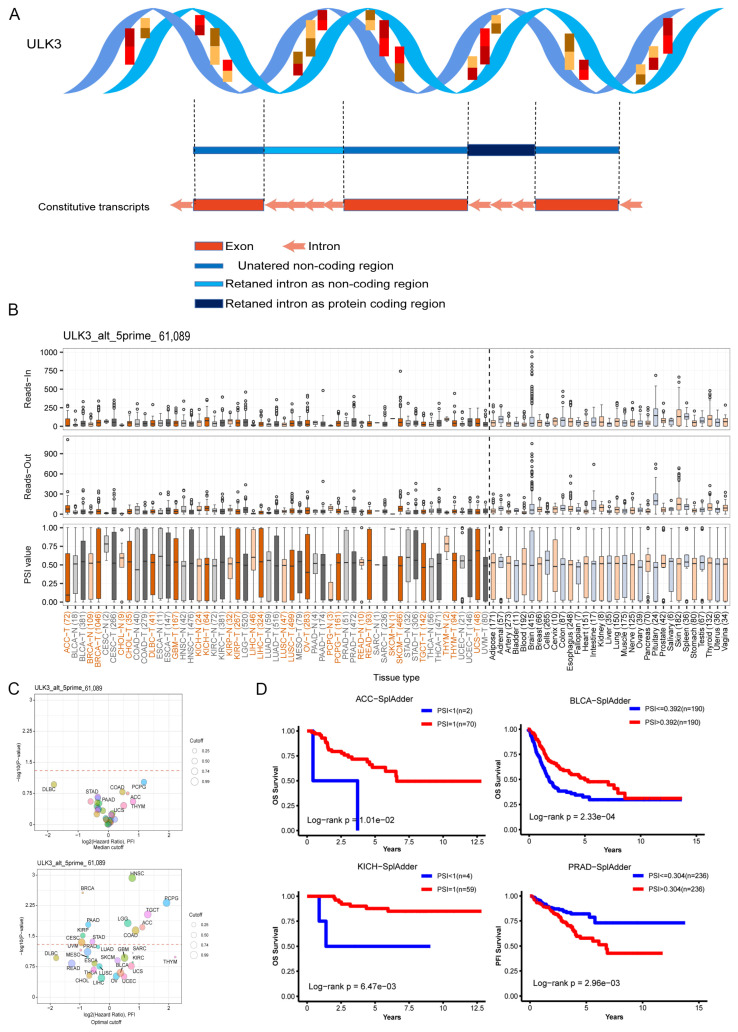
*ULK3* alternative splicing is associated with patient prognosis. (**A**) A schematic diagram illustrates the *ULK3* alternative splicing event, designated *ULK3*_alt_5prime_61089. (**B**) Reads-in, reads-out, and percent-spliced-in (PSI) values for *ULK3*_alt_5prime_61089 are shown across pan-cancer tumor tissues, matched adjacent non-tumor tissues, and healthy normal tissues. Cancers and their corresponding adjacent tissues are labeled in distinct colors; non-tumor tissues from healthy donors are labeled in black. (**C**) PSI values are compared between tumor and matched adjacent tissues (top panel) and between tumor and GTEx-derived normal tissues (bottom panel). The red dashed line indicates a false discovery rate (FDR) threshold of 0.05. Dot size reflects the tumor PSI value, and each cancer type is color-coded accordingly. (**D**) Kaplan–Meier curves depict overall survival (OS) stratified by *ULK3*_alt_5prime_61089 PSI levels. All data were obtained from the OncoSplicing online resource. The red and blue curves represent high-PSI and low-PSI patient subgroups, respectively: the blue curve corresponds to patients with low splicing inclusion (low PSI), whereas the red curve corresponds to patients with high splicing inclusion (high PSI).

The impact of alternative splicing on cancer progression has been well recognized. Using the OncoSplice platform, we identified several *ULK3* splicing variants. Figure 5A illustrates the *ULK3*-ALT-5-prist-61089 splicing event. This panel shows its splice-site architecture and cancer type-specific distribution pattern. Figure 5B shows the percent spliced-in (PSI) values of this splicing event in cancer tissues and normal tissues (Supplementary Table S6). This panel also presents the read-out and read-in data. Notably, the PSI values in BLCA, BRCA, COAD, HNSC, KICH, KIRC, KIRP, PCPG, READ, and THCA were higher than those in normal tissues. Figure 5C stratifies survival prognosis according to the median and optimal cutoff values of *ULK3*-ALT-5-prist-61089 expression. The results showed significant differences in kidney renal clear cell carcinoma and breast cancer. The Kaplan–Meier survival curves in Figure 5D showed that high PSI values in ACC, BLCA, and KICH were associated with shorter overall survival (OS). High PSI values in PRAD were associated with shorter progression-free interval (PFI). These results suggest that dysregulated *ULK3* alternative splicing may play an important role in determining clinical outcomes in cancer patients.

### 2.5. Enrichment Evaluation of Genes Co-Expressed with ULK3 in a Range of Tumors

**Figure 6:**
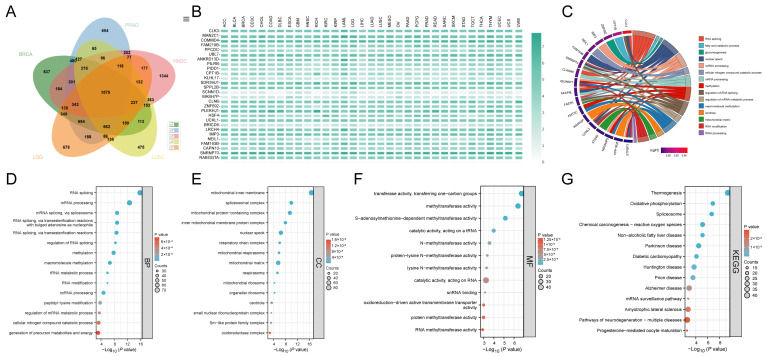
Enrichment analysis of *ULK3* co-expressed genes in various tumors. (**A**) A Venn diagram generated by SangerBox shows the overlap of *ULK3* co-expressed genes in five different tumor types: BRCA (green), LGG (orange), LUSC (yellow), HNSC (red), and PRAD (blue). (**B**) A heat map generated by Gene Expression Profiling Interactive Analysis 2.0 (GEPIA2.0) shows the expression levels of 30 *ULK3* co-expressed genes across various cancers. The color gradient from light green to dark green indicates increasing gene expression levels. (**C**) A Gene Ontology (GO) circle diagram generated through the Science and Research Online Plot (SRplot) shows the enrichment of these 838 *ULK3* co-expressed genes. Biological process pathways are highlighted with colored bands, and the band width corresponds to the number of enriched genes. (**D**) Biological processes associated with *ULK3* co-expressed genes. (**E**) Cellular components associated with these genes. (**F**) Molecular functions of *ULK3* co-expressed genes. (**G**) KEGG pathway analysis shows significant enrichment. The size of each bubble corresponds to the number of genes involved, and the color intensity indicates the significance level (*p*-value).

To explore the biological relevance of *ULK3* and its co-expressed genes in cellular functions, we performed Gene Ontology (GO) term and Kyoto Encyclopedia of Genes and Genomes (KEGG) pathway enrichment analyses. These analyses were used to clarify the potential impact of *ULK3*-related genes on cancer progression. GO and KEGG enrichment analyses are essential approaches for elucidating the functional roles of genes in biological processes and cellular mechanisms. Based on the above results, BRCA, LUSC, PRAD, LGG, and HNSC showed high *ULK3* mRNA expression, stemness features, high levels of RNA modification, and poor patient survival. These five cancers also represent distinct tissue origins, including breast, lung, prostate, brain, and head and neck tissues. Therefore, they allowed us to identify *ULK3*-associated transcriptional programs that may extend beyond tissue-specific effects. We sought to determine whether common *ULK3* co-expressed genes could be identified across these cancers. We also assessed their potential contribution to critical ULK3-related functions in tumorigenesis. We first downloaded *ULK3*-positive co-expressed genes in these five cancers from cBioPortal. We then performed Venn diagram analysis using the SangerBox platform and identified 1579 shared genes among the five cancer types examined (Figure 6A). Next, we examined the mRNA expression profiles of these 1579 shared genes across cancer types. The heat map showed that 30 of these genes were highly expressed in multiple cancers (Figure 6B). This finding suggested that these genes may play important roles in organismal development and cancer progression. GO enrichment analysis showed that these genes were associated with several biological processes. These processes included precursor metabolite generation, energy production, RNA splicing, mRNA processing, regulation of RNA splicing, methylation, macromolecular methylation, RNA modification, non-coding RNA processing, and cellular nitrogen compound catabolism (Figure 6C,D). Cellular component analysis showed that these genes were mainly localized in mitochondria, nuclei, ribosomes, and neutrophil-related structures (Figure 6E). Molecular function enrichment analysis showed that these genes were associated with one-carbon group transferase activity, methyltransferase activity, S-adenosylmethionine-dependent methyltransferase activity, tRNA-directed catalytic activity, N-methyltransferase activity, and lysine/protein-lysine N-methyltransferase activity. Many of these functions are related to RNA regulation. These genes were also enriched in catalytic activity, rRNA binding, oxidoreductase-driven active transmembrane transporter activity, protein methyltransferase activity, and RNA methyltransferase activity (Figure 6F). KEGG pathway enrichment analysis further showed that these genes were closely associated with thermogenesis, oxidative phosphorylation, chemical carcinogenesis-reactive oxygen species, non-alcoholic fatty liver disease, diabetic cardiomyopathy, mRNA surveillance pathways, and multiple neurodegenerative disease pathways. These neurodegenerative disease pathways included Alzheimer’s disease, Parkinson’s disease, Huntington’s disease, amyotrophic lateral sclerosis, and prion disease (Figure 6G). Taken together, these results indicate that *ULK3* co-expressed genes are involved in core biological processes, including energy metabolism, RNA post-transcriptional modification and processing, methylation, and catabolism. These genes may contribute to the pathogenesis of metabolic disorders, cancers, and various neurodegenerative diseases by modulating key pathways, such as oxidative phosphorylation, oxidative stress responses, and mRNA homeostasis.

### 2.6. The Link Between ULK3, Cancer Stem Cells, and RNA Modification

**Figure 7:**
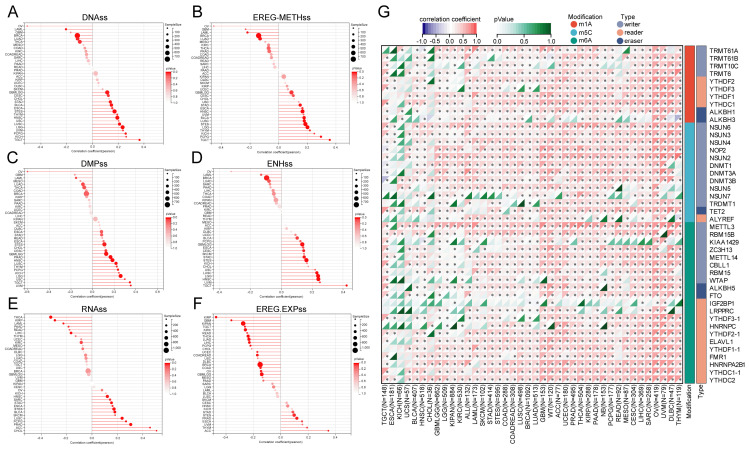
Correlation of *ULK3* with tumor stemness and RNA modification. (**A**–**F**) Lollipop plots show the correlations between *ULK3* and stemness indices, including DNAss, EREG-METHss, DMPss, ENHss, RNAss, and EREG-EXPss. (**G**) A heat map shows the correlations between *ULK3* and RNA modifier genes, including *m1A*, *m5C*, and *m6A* regulators. Colors indicate the strength of the correlation, with red indicating positive correlations and blue indicating negative correlations. *p*-values and significance levels (*) are labeled in the figure. Pearson correlation analysis was performed using the corr. test function from the R package psych v2.1.6, with FDR correction for multiple comparisons. * *p* < 0.05.

We assessed the relationship between *ULK3* and tumor stemness indices across multiple cancer types. Significant associations were observed in several tumor types. Across multiple stemness indices, including DNAss, EREG-METHss, DMPss, ENHss, RNAss, and EREG-EXPss, *ULK3* showed notable positive correlations with these indices in ESCA, PRAD, stomach and esophageal carcinoma (STES), THYM, and ACC. These results indicate that ULK3 may help maintain the stemness phenotype of tumor cells. ULK3 may also facilitate invasion and confer resistance to therapy. In contrast, *ULK3* showed strong negative correlations with stemness indices in tumors such as LAML, BRCA, and LUAD (Figure 7A–F, Supplementary Table S7). We further found that *ULK3* expression was correlated with promoter methylation status. Tumors with high *ULK3* expression also showed RNA profiles resembling those of stem cells. In most tumor types, *ULK3* mRNA expression was significantly and positively correlated with m1A, m5C, and m6A RNA-modifying enzymes. In contrast, *ULK3* showed a negative correlation with these RNA modification regulators in TGCT (Figure 7G). These results suggest that RNA modification may be involved in *ULK3*-related mechanisms of tumor progression. This study indicates that ULK3 may serve as a potential marker of tumor stemness and may provide a new therapeutic target for aggressive disease phenotypes.

### 2.7. Association Analysis Between ULK3 and Immune Infiltration in the Tumor Microenvironment

**Figure 8:**
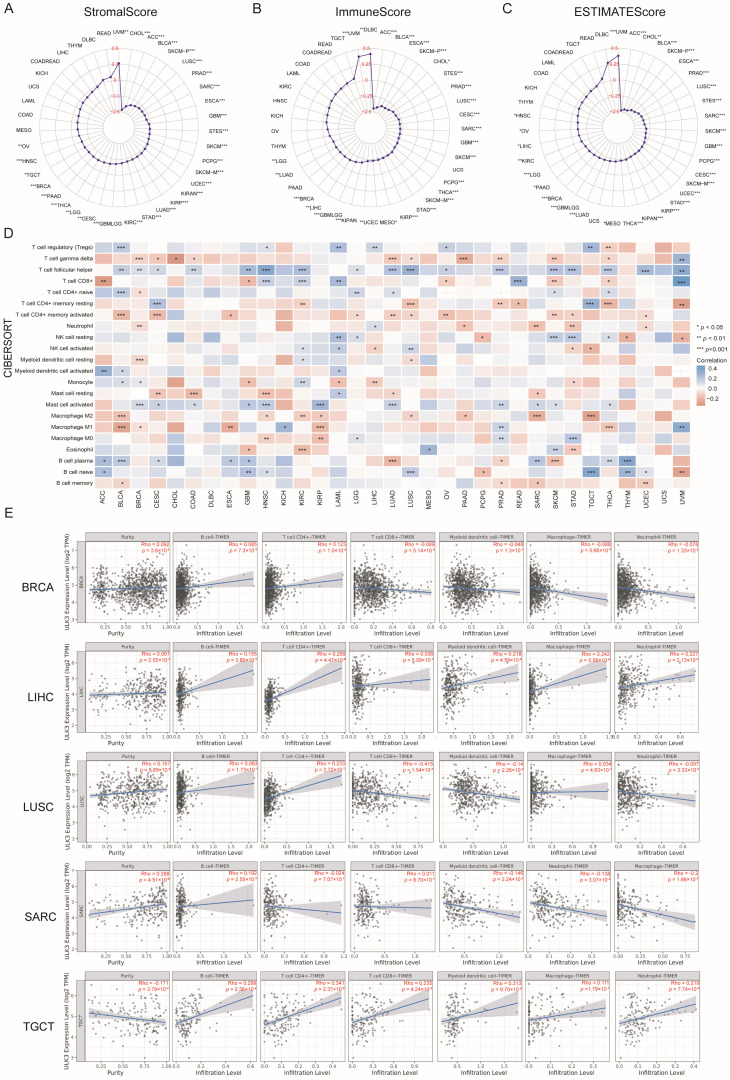
Correlation analysis between *ULK3* expression and immune cell infiltration in various tumors. Radar plots show the correlations between *ULK3* expression and immune-related scores, including Stromal Score (**A**), Immune Score (**B**), and ESTIMATE Score (**C**), across different cancers. These analyses were performed using SangerBox. Cancer types are labeled around the radar plots. Pearson correlation analysis was performed using the corr. test function from the R package psych v2.1.6, with FDR correction. (**D**) CIBERSORT was used to analyze the correlations between *ULK3* expression and the infiltration abundance of 22 immune cell subsets, including Tregs, CD8+ T cells, and macrophages, across multiple cancers. In the heatmap, blue denotes positive correlation, red denotes negative correlation, and darker shading reflects stronger correlation. * *p* < 0.05, ** *p* < 0.01, *** *p* < 0.001. (**E**) TIMER2.0 was used to analyze the correlations between *ULK3* expression and the infiltration abundance of immune cell subsets, including B cells, CD4+ T cells, and CD8+ T cells, in BRCA, LIHC, LUSC, SARC, and TGCT. The gray shaded area along the blue fitted curve (trend line) represents the 95% confidence interval of the correlation fit. Spearman partial correlation analysis was performed after adjustment for tumor purity, with FDR correction.

Within the tumor microenvironment (TME), multiple cell types, including immune cells and stromal cells, coexist with extracellular components. Immune infiltration profiling provides a detailed view of the cellular composition and activation status of immune populations within the TME. It also reflects the capacity of the TME to either promote or suppress tumor growth and metastasis. Stromal Score quantifies stromal cells, such as fibroblasts and mesenchymal cells, and reflects the connective tissue compartment that provides structural support within the TME. Tumors with abundant stromal components tend to show more aggressive phenotypes, greater resistance to therapy, and poorer prognosis. Immune Score estimates the abundance of infiltrating immune cell populations in the TME, especially lymphocytes such as T cells, B cells, and natural killer (NK) cells. A higher Immune Score is generally associated with improved responsiveness to immunotherapy and better survival outcomes. ESTIMATE Score is a composite score derived from Stromal Score and Immune Score. A higher ESTIMATE Score indicates a poorer response to immunotherapy. We investigated whether *ULK3* expression was associated with immune infiltration patterns and patient responses to immunotherapy. Immune-related scores were calculated using raw data obtained from SangerBox. Our findings showed a strong positive correlation between *ULK3* expression and Stromal Score in UVM (R = 0.32, *p* = 4.6 × 10^−3^) and LAML (R = 0.27, *p* = 5.1 × 10^−5^). In contrast, *ULK3* expression was significantly negatively correlated with Stromal Score in GBM (R = −0.33, *p* = 3.8 × 10^−5^), GBMLGG (R = −0.21, *p* = 3.7 × 10^−8^), LGG (R = −0.19, *p* = 2.5 × 10^−5^), UCEC (R = −0.27, *p* = 3.5 × 10^−4^), BRCA (R = −0.17, *p* = 2.4 × 10^−8^), CESC (R = −0.20, *p* = 7.1 × 10^−4^), LUAD (R = −0.25, *p* = 1.8 × 10^−8^), ESCA (R = −0.36, *p* = 7.3 × 10^−7^), STES (R = −0.32, *p* = 1.1 × 10^−14^), SARC (R = −0.36, *p* = 2.3 × 10^−9^), KIRP (R = −0.25, *p* = 1.7 × 10^−5^), the pan-kidney cohort KIPAN (R = −0.26, *p* = 1.5 × 10^−14^), PRAD (R = −0.37, *p* = 2.8 × 10^−17^), STAD (R = −0.24, *p* = 1.2 × 10^−6^), HNSC (R = −0.15, *p* = 5.1 × 10^−4^), KIRC (R = −0.23, *p* = 1.3 × 10^−7^), LUSC (R = −0.37, *p* = 1.2 × 10^−17^), SKCM-P (R = −0.39, *p* = 4.4 × 10^−5^), SKCM (R = −0.31, *p* = 1.3 × 10^−11^), BLCA (R = −0.42, *p* = 5.2 × 10^−19^), SKCM-M (R = −0.28, *p* = 1.4 × 10^−7^), THCA (R = −0.17, *p* = 8.5 × 10^−5^), NB (R = −0.22, *p* = 5.2 × 10^−3^), OV (R = −0.13, *p* = 6.2 × 10^−3^), PAAD (R = −0.17, *p* = 0.02), PCPG (R = −0.30, *p* = 5.0 × 10^−5^), ACC (R = −0.48, *p* = 1.2 × 10^−5^), ALL-R (R = −0.24, *p* = 0.02), and CHOL (R = −0.56, *p* = 3.9 × 10^−4^) (Figure 8A, Supplementary Table S8). For Immune Score, *ULK3* expression was positively correlated with Immune Score in UVM (R = 0.38, *p* = 4.7 × 10^−4^) and DLBC (R = 0.41, *p* = 4.6 × 10^−3^). In contrast, *ULK3* expression was significantly negatively correlated with Immune Score in GBM (R = −0.28, *p* = 5.7 × 10^−4^), GBMLGG (R = −0.17, *p* = 2.1 × 10^−5^), LGG (R = −0.13, *p* = 3.5 × 10^−3^), UCEC (R = −0.22, *p* = 2.8 × 10^−3^), BRCA (R = −0.14, *p* = 2.0 × 10^−6^), CESC (R = −0.28, *p* = 8.5 × 10^−7^), LUAD (R = −0.13, *p* = 3.7 × 10^−3^), ESCA (R = −0.37, *p* = 3.1 × 10^−7^), STES (R = −0.31, *p* = 1.9 × 10^−14^), SARC (R = −0.28, *p* = 4.6 × 10^−6^), KIRP (R = −0.23, *p* = 9.2 × 10^−5^), KIPAN (R = −0.19, *p* = 2.9 × 10^−8^), PRAD (R = −0.13, *p* = 3.6 × 10^−12^), STAD (R = −0.23, *p* = 3.6 × 10^−6^), LUSC (R = −0.29, *p* = 1.1 × 10^−10^), LIHC (R = −0.15, *p* = 4.3 × 10^−3^), WT (R = −0.27, *p* = 0.02), SKCM-P (R = −0.37, *p* = 1.7 × 10^−4^), SKCM (R = −0.28, *p* = 2.5 × 10^−9^), BLCA (R = −0.42, *p* = 2.1 × 10^−18^), SKCM-M (R = −0.24, *p* = 8.1 × 10^−6^), THCA (R = −0.24, *p* = 8.3 × 10^−8^), MESO (R = −0.23, *p* = 0.04), PCPG (R = −0.26, *p* = 5.3 × 10^−4^), ACC (R = −0.45, *p* = 4.6 × 10^−5^), ALL-R (R = −0.26, *p* = 0.01), and CHOL (R = −0.35, *p* = 0.04) (Figure 8B, Supplementary Table S8). *ULK3* expression showed a strong positive correlation with ESTIMATE Score in UVM (R = 0.38, *p* = 5.3 × 10^−4^) and LAML (R = 0.14, *p* = 0.04) (Figure 8C). In contrast, *ULK3* expression was significantly negatively correlated with ESTIMATE Score in GBM (R = −0.31, *p* = 1.0 × 10^−4^), GBMLGG (R = −0.19, *p* = 1.0 × 10^−6^), LGG (R = −0.16, *p* = 4.4 × 10^−4^), UCEC (R = −0.26, *p* = 4.0 × 10^−4^), BRCA (R = −0.18, *p* = 7.0 × 10^−9^), CESC (R = −0.28, *p* = 1.5 × 10^−6^), LUAD (R = −0.20, *p* = 7.7 × 10^−6^), ESCA (R = −0.40, *p* = 3.6 × 10^−8^), STES (R = −0.34, *p* = 6.7 × 10^−17^), SARC (R = −0.34, *p* = 3.4 × 10^−8^), KIRP (R = −0.25, *p* = 1.7 × 10^−5^), KIPAN (R = −0.23, *p* = 4.3 × 10^−12^), PRAD (R = −0.36, *p* = 7.2 × 10^−17^), STAD (R = −0.26, *p* = 2.0 × 10^−7^), HNSC (R = −0.11, *p* = 0.01), KIRC (R = −0.13, *p* = 2.7 × 10^−3^), LUSC (R = −0.35, *p* = 3.0 × 10^−15^), LIHC (R = −0.12, *p* = 0.02), WT (R = −0.25, *p* = 0.02), SKCM-P (R = −0.41, *p* = 2.0 × 10^−5^), SKCM (R = −0.31, *p* = 1.5 × 10^−11^), BLCA (R = −0.45, *p* = 2.3 × 10^−21^), SKCM-M (R = −0.27, *p* = 2.9 × 10^−7^), THCA (R = −0.23, *p* = 2.6 × 10^−7^), MESO (R = −0.21, *p* = 0.05), OV (R = −0.11, *p* = 0.02), PAAD (R = −0.16, *p* = 0.03), PCPG (R = −0.30, *p* = 6.7 × 10^−5^), ACC (R = −0.48, *p* = 1.1 × 10^−5^), ALL-R (R = −0.27, *p* = 6.9 × 10^−3^), and CHOL (R = −0.45, *p* = 5.6 × 10^−3^) (Figure 8C, Supplementary Table S8). Taken together, these results support the potential role of *ULK3* as a predictor of tumor sensitivity to various treatments. To further explore the contribution of *ULK3* at the immune cell level and its potential value as an immune-related marker in cancer, we used the CIBERSORT algorithm to analyze correlations between *ULK3* expression and 22 immune cell subsets. The key finding was that *ULK3* expression was positively correlated with M2 macrophages in eight cancer types, including BLCA, HNSC, KIRC, KIRP, LUSC, PAAD, SARC, and TGCT. By comparison, *ULK3* expression was negatively associated with regulatory T cells (Tregs) in six distinct tumor types (Figure 8D). This integrative analysis supported a robust correlation between *ULK3* expression and M2 macrophage infiltration. These findings provide evidence for further investigation of the role of macrophages in cancer.

We further investigated additional components of the tumor immune microenvironment that contribute to immunosuppression. Using TIMER2.0, we analyzed the relationship between *ULK3* expression and immunosuppressive cells, including B cells, CD4+ T cells, CD8+ T cells, myeloid dendritic cells (MDCs), macrophages, and neutrophils (NE). Significant associations with at least two distinct immune cell types were confirmed in BRCA, LIHC, LUSC, SARC, and TGCT. After adjusting for tumor purity as a covariate, *ULK3* showed the strongest association with NE (Figure 8E).

### 2.8. The Function of ULK3 in Immune Modulation and Tumor Infiltration

**Figure 9:**
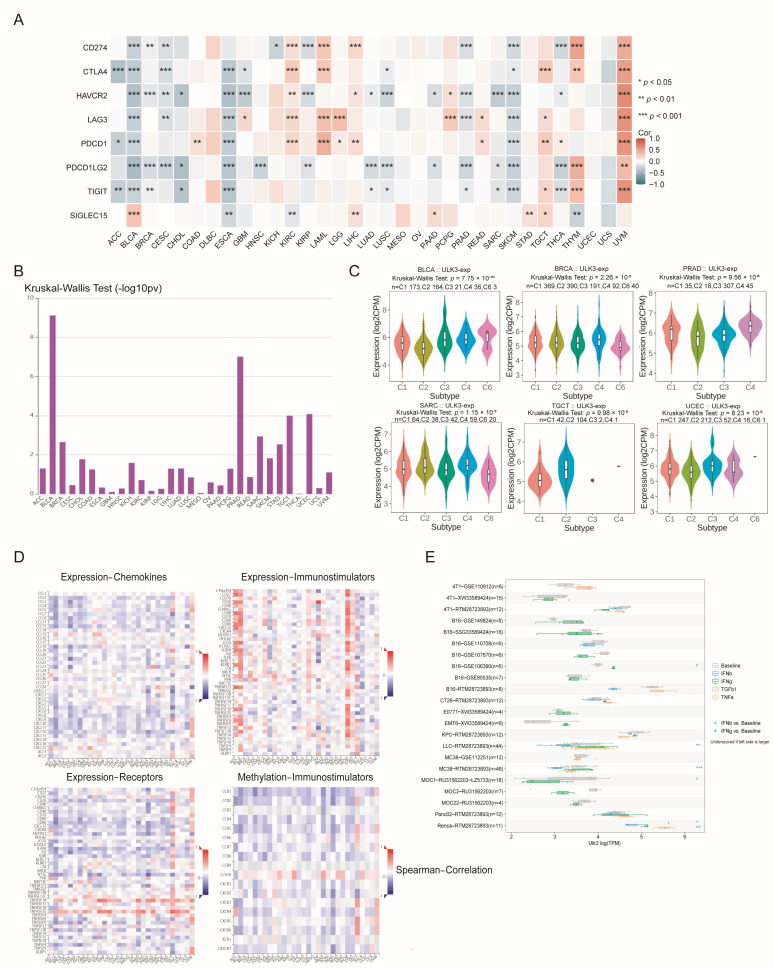
Relationship between *ULK3* and immune infiltration and cytokines. (**A**) This panel shows the association between immune checkpoint genes and *ULK3* expression. Pearson correlation analysis was performed with FDR correction. (**B**) Correlations between *ULK3* and immune subtypes were obtained from the TISIDB online tool. The unpaired Wilcoxon rank-sum test was used with FDR correction. (**C**) This panel shows *ULK3* expression in six immune subtypes across six different cancer types. The unpaired Wilcoxon rank-sum test was used with FDR correction. (**D**) The heat maps show the correlations between *ULK3* expression and chemokines (**top left**), receptors (**bottom left**), and immune activators (**top right**). In addition, a heat map shows the correlation between *ULK3* promoter methylation levels and immune activators (**bottom right**). Pearson correlation analysis was performed with FDR correction. (**E**) This panel shows *ULK3* expression in cancer cell lines before and after cytokine treatment. The paired *t*-test was used. * *p* < 0.05, ** *p* < 0.01, *** *p* < 0.001.

We further assessed the potential regulatory involvement of *ULK3* in immune checkpoints by co-expression profiling across 33 cancer types. As shown in the heat map, *ULK3* was co-expressed with most immune checkpoint genes across cancers. However, this pattern was not observed in CHOL, DLBC, KICH, MESO, OV, PAAD, READ, UCEC, and UCS (*p* < 0.05) (Figure 9A). Among these associations, strong positive correlations with CD274, *CTLA4*, and *LAG3* were observed in 25 cancers, especially in BLCA. These findings highlight the potential role of *ULK3* in immune checkpoint regulation. Based on data from the Tumor–Immune System Interactions and DrugBank database (TISIDB), we systematically evaluated *ULK3* expression across multiple immune subtypes. Statistically significant associations were detected in nine cancer types. The most significant correlations, including those in BLCA, BRCA, PRAD, SARC, TGCT, and UCEC, are presented as bar graphs (Figure 9B) and are shown in detail in Figure 9C. According to the heat map shown in Figure 9D, *ULK3* expression was inversely associated with immunostimulatory, chemokines, and their receptors in several tumor types. Finally, we used the Tumor Immune Syngeneic Mouse (TISMO) database to compare *ULK3* expression levels in multiple cancer cell lines before and after cytokine exposure. The results showed that IFN-β treatment decreased *ULK3* expression in three cell lines. IFN-γ treatment caused similar reductions in all three cell lines. In contrast, TNF-α treatment did not reduce *ULK3* expression in these cell lines (Figure 9E). These findings indicate that ULK3 may contribute to the immunosuppressive environment in multiple cancers by interacting with immunostimulatory and regulating immune checkpoint mechanisms. This multidimensional analysis provides important insights into the complex mechanisms through which ULK3 may influence immune regulation and cancer progression.

### 2.9. Analysis of the Association Between ULK3 Expression and Immunotherapy Biomarkers

**Figure 10:**
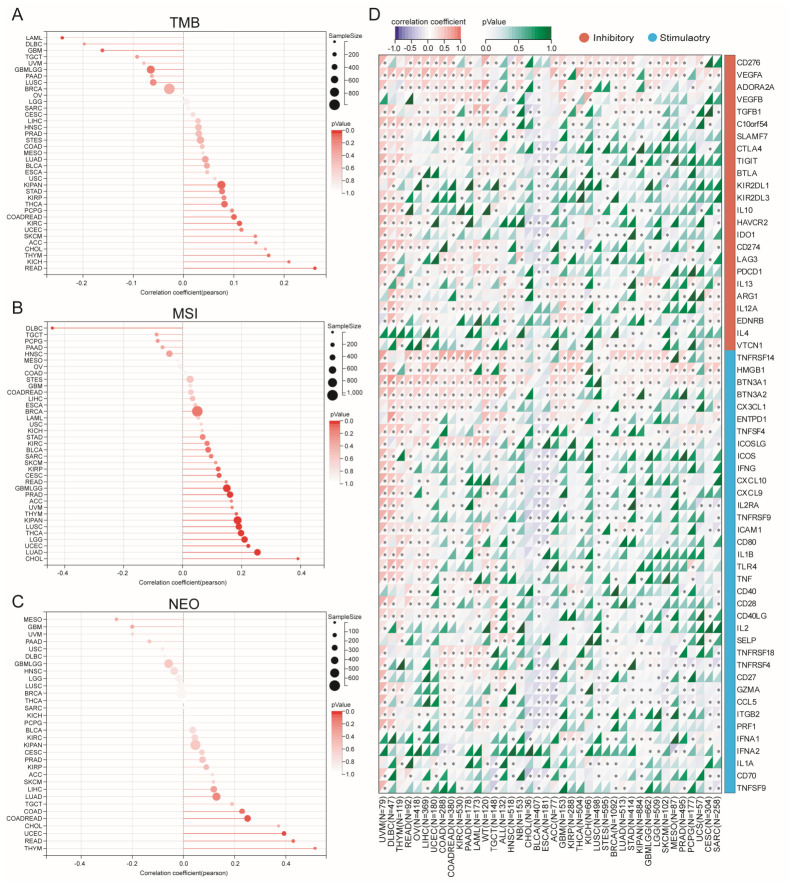
Correlation analysis between *ULK3* and immune checkpoint blockade-related proteins. (**A**–**C**) Lollipop plots generated by SangerBox show the correlations between *ULK3* expression and TMB (**A**), MSI (**B**), and NEO (**C**) across multiple cancers. The horizontal axis represents Pearson’s correlation coefficient. Positive values indicate positive correlations, and negative values indicate negative correlations. Bubble size corresponds to sample size. Bubble color represents the *p*-value of the correlation; redder colors indicate smaller *p*-values and stronger statistical significance. The red horizontal line represents the confidence interval of the correlation coefficient. Pearson correlation analysis was performed with FDR correction. (**D**) A heat map generated by SangerBox shows the correlations between *ULK3* expression and multiple immune checkpoint blockade-related genes across multiple cancer types. Pearson correlation analysis was performed with FDR correction. * *p* < 0.05. The color gradient indicates the correlation coefficient, with red indicating a positive correlation and blue indicating a negative correlation. Significant correlations are indicated by asterisks, and dark green indicates nonsignificant correlations.

We further investigated whether *ULK3* expression was correlated with tumor mutational burden (TMB), microsatellite instability (MSI) status, and neoantigen (NEO) abundance. These analyses were performed to clarify the potential association of *ULK3* with neoantigen production and immunotherapy response. *ULK3* expression showed significant positive correlations with TMB in KIPAN (R = 0.075, *p* = 4.98 × 10^−2^), KIRC (R = 0.111, *p* = 4.21 × 10^−2^), and READ (R = 0.261, *p* = 1.28 × 10^−2^). These findings suggest an association between increased *ULK3* expression and elevated mutation counts in these tumors. In contrast, *ULK3* expression showed significant negative correlations with TMB in GBM (R = −0.161, *p* = 4.93 × 10^−2^) and LAML (R = −0.241, *p* = 7.21 × 10^−3^), indicating an inverse relationship between *ULK3* expression and mutation burden in these tumor types (Figure 10A, Supplementary Table S9). *ULK3* expression was significantly positively correlated with MSI in GBMLGG (R = 0.149, *p* = 1.17 × 10^−4^), LGG (R = 0.209, *p* = 1.93 × 10^−6^), CESC (R = 0.123, *p* = 3.20 × 10^−2^), LUAD (R = 0.254, *p* = 6.13 × 10^−9^), KIRP (R = 0.120, *p* = 4.24 × 10^−2^), KIPAN (R = 0.186, *p* = 8.71 × 10^−7^), PRAD (R = 0.161, *p* = 3.34 × 10^−4^), UCEC (R = 0.223, *p* = 2.67 × 10^−3^), LUSC (R = 0.190, *p* = 2.19 × 10^−5^), THYM (R = 0.182, *p* = 4.86 × 10^−2^), THCA (R = 0.198, *p* = 9.64 × 10^−6^), and CHOL (R = 0.391, *p* = 1.83 × 10^−2^). These results suggest that patients with higher *ULK3* expression may be more likely to respond to immunotherapy in these tumor types. In contrast, *ULK3* expression showed a significant inverse correlation with MSI in DLBC (R = −0.442, *p* = 1.87 × 10^−3^). This finding suggests that lower *ULK3* expression may be associated with a reduced likelihood of immunotherapy benefit in this cancer type (Figure 10B, Supplementary Table S9). For NEO, *ULK3* expression showed significant positive correlations in COADREAD (R = 0.125, *p* = 2.94 × 10^−2^) and UCEC (R = 0.196, *p* = 1.63 × 10^−2^) (Figure 10C, Supplementary Table S9). These findings indicate that immunotherapy may yield better outcomes in patients with elevated *ULK3* expression in these cancers.

We next investigated the association between *ULK3* and immune checkpoint proteins, given their roles as targets of checkpoint blockade therapy. *ULK3* showed marked positive associations with most immune checkpoint blockade-related proteins in UVM, DLBC, OV, THYM, and READ. These results suggest that *ULK3* may be involved in modulating tumor immune evasion. Therefore, patients with high *ULK3* expression may be more likely to benefit from checkpoint inhibitor therapy in these cancers. In contrast, *ULK3* showed marked inverse associations with most immune checkpoint proteins in BLCA, ESCA, and CHOL (Figure 10D), suggesting that *ULK3* may be associated with more active antitumor immunity in these tumor types.

### 2.10. Subcellular Localization of ULK3 and Its Cancer-Promoting Function in Prostate Cancer Cells

**Figure 11:**
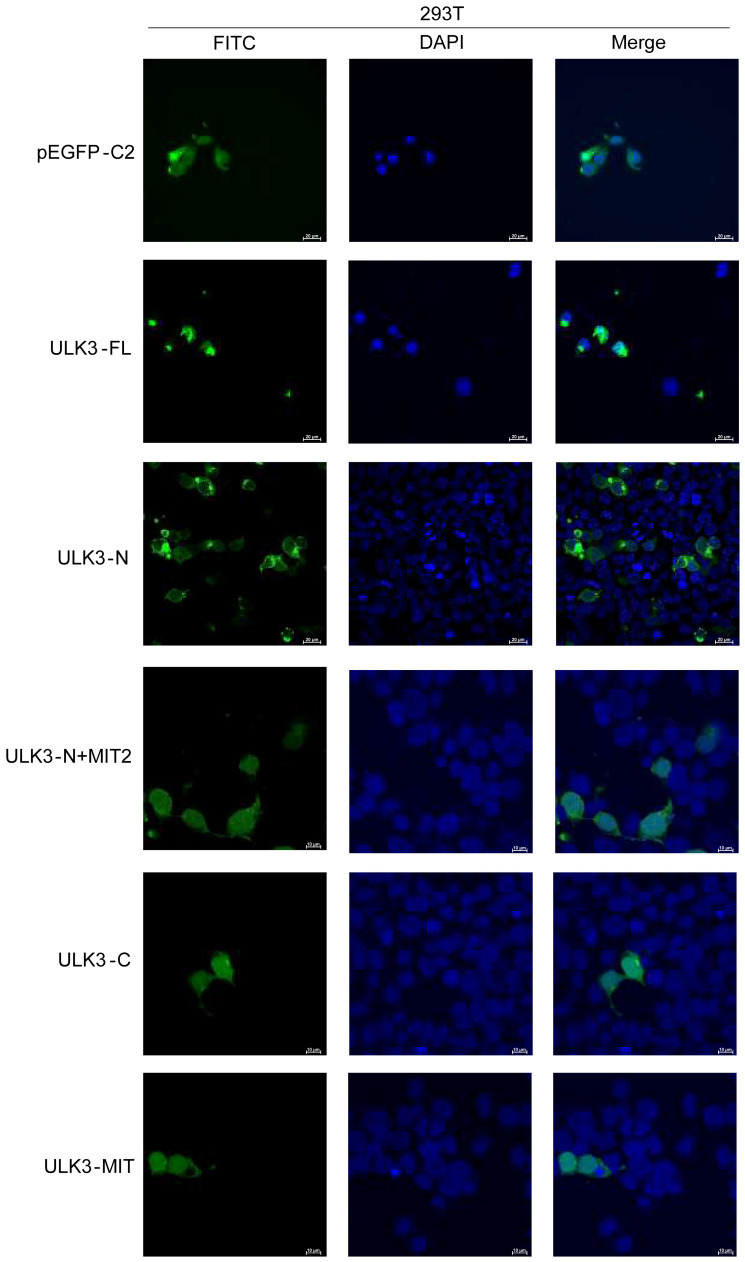
Subcellular localization of full-length ULK3 and its truncated proteins in 293T cells. pEGFP-C2: empty vector (mock); ULK3-FL: ULK3-full length; ULK3-N: Serine/Threonine protein kinase, catalytic domain (STKc); ULK3-N + MIT2: STKc domain and Microtubule Interacting and Trafficking domain 2 (MIT2); ULK3-MIT: Microtubule Interacting and Trafficking domain (MIT); and ULK3-C: MIT and MIT2 domains. Scale bars: 10 μm and 20 μm.

The pEGFP-C2 empty vector, *ULK3*-FL plasmid, and truncated *ULK3* plasmids were transfected into 293T cells. DAPI staining was performed to visualize the nuclei, and green fluorescence was observed to determine the expression and subcellular localization of exogenous ULK3 protein and its domains in eukaryotic cells. The results showed that fluorescence from the pEGFP-C2 empty vector was mainly distributed in the cytoplasm, with partial localization in the nucleus. Full-length ULK3, ULK3-N + MIT2, ULK3-MIT, and ULK3-C were detected in both the nucleus and cytoplasm. ULK3-N was mainly localized in the cytoplasm and was partially detected in the nucleus. These results suggest that different domains of ULK3 may have distinct cellular roles (Figure 11).

**Figure 12:**
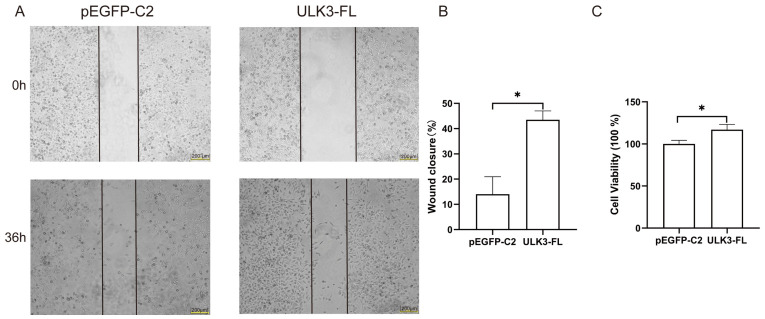
Migration and proliferation abilities of PC-3 cells after *ULK3* overexpression. (**A**,**B**) The migration ability of PC-3 cells was detected after *ULK3* overexpression. (**C**) Cell viability of PC-3 cells was detected after *ULK3* overexpression. The experiments were independently repeated three times. Statistical analysis was performed using a two-sided Student’s *t*-test. * *p* < 0.05.

PC-3 cells were transfected with the pEGFP-C2 empty vector or the full-length *ULK3*-FL plasmid. The effect of *ULK3* overexpression on PC-3 cell migration was evaluated by recording wound closure at 0 h and 36 h after scratching. The results showed that the wound-healing capacity of PC-3 cells overexpressing *ULK3* was significantly greater than that of control cells (*p* < 0.05). These findings indicate that *ULK3* overexpression promoted the migration of PC-3 cells (Figure 12A,B). PC-3 cells were also transfected with the pEGFP-C2 empty vector or the full-length *ULK3*-FL plasmid, and cell viability was measured after 48 h. The MTT assay showed that the viability of PC-3 cells overexpressing *ULK3* was significantly higher than that of the control group (*p* < 0.05). These results indicate that *ULK3* overexpression promoted the proliferation of PC-3 cells (Figure 12C).

### 2.11. Discovery of Drugs That Activate ULK3

**Figure 13:**
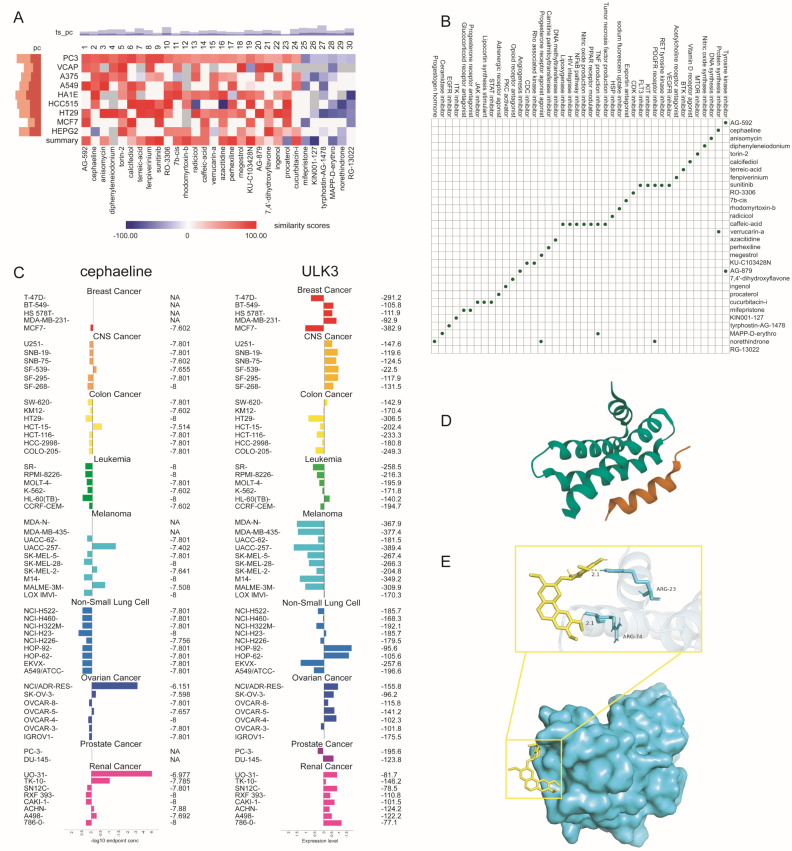
Identification of ULK3-activating drugs and molecular binding analysis. (**A**) Heat map evaluation of the top 30 compounds screened based on the most upregulated and downregulated genes associated with ULK3. The similarity score is indicated by color. PC represents the percentage of total perturbation signatures based on the number of rows above a given threshold across all column samples. The height of the dark orange bar indicates a connection rate ≥ 97.5. ts_pc indicates the total percentage of gold-standard perturbagens connected to the selected row above a specified threshold. The height of the dark blue bar indicates a connection percentage ≥ 97.5. (**B**) MoA scatter plot of the top 30 compounds shown in panel (**A**). (**C**) Evaluation of −log10(GI50) values (**left**) and ULK3 expression levels (**right**) in cell lines. The center line corresponds to the mean −log10(GI50) value or the mean ULK3 expression value. Each color represents a cancer type. (**D**) Three-dimensional structure model of ULK3. (**E**) The image shows the three-dimensional structure of ULK3 used for drug binding analysis in blue. The two-dimensional structural features of the candidate drug are shown in yellow. Interacting amino acid residues, molecular forces, and molecular distances are also presented. Abbreviation: Arg, arginine.

It is important to identify drugs capable of activating ULK3. Using the Connectivity Map (CMap) tool to screen candidate compounds, we identified 30 potential ULK3-stimulatory compounds based on consistent transcriptomic changes across nine cell lines (Figure 13A). These compounds suggested a close association between ULK3 and distinct molecular mechanisms in tumor cells (Figure 13B). In addition, we used the COMPARE method to evaluate GI50 values for these compounds across diverse cancer cell lines. NM-PPI was excluded because of the limited availability of testing data. Cephaeline has not been reported in prostate cancer cells, supporting its novelty as a candidate compound for prostate cancer treatment (Figure 13C). To evaluate the binding potential between ULK3 protein and Cephaeline, molecular docking studies were performed. The results showed that Cephaeline could successfully dock with ULK3 (Figure 13D,E). These findings indicate that Cephaeline may be a promising candidate compound for ULK3 activation.

## 3. Discussion

Cancer initiation and progression are largely driven by dysregulated molecular programs, including abnormal gene expression. These abnormalities may manifest as altered transcriptional levels, dysregulated post-translational modifications, or epigenetic changes. Such alterations not only promote malignant transformation but also affect patient prognosis by regulating the tumor microenvironment (TME), immune escape, and metabolic reprogramming [11]. In this study, we integrated TCGA, GTEx, and other public databases to analyze the expression profile, genomic variation, clinical prognostic relevance, immune microenvironment, and potential biological functions of *ULK3* across 33 cancer types. Our results showed that *ULK3* was dysregulated in most cancer types. Its aberrant expression was significantly associated with various clinicopathological features and poor prognosis. Bioinformatics analyses suggested potential correlations with RNA modification, energy metabolism, and TME remodeling. However, these mechanistic links still require experimental validation. These findings suggest that ULK3 may serve as a broadly relevant tumor biomarker and a potential therapeutic target.

Consistent upregulation of ULK3 mRNA and protein was detected in 13 malignancies, including BLCA, BRCA, COAD, LIHC, LUAD, and LUSC. In contrast, *ULK3* expression was decreased only in CHOL and HNSC. This broad dysregulation pattern suggests that *ULK3* may show either tumor-promoting or tumor-suppressive correlation patterns in different cancer types. This finding indicates context-dependent functional heterogeneity. Notably, the prognostic significance of *ULK3* varied across cancer types. High *ULK3* expression was associated with poor survival outcomes in ACC, KIRC, SKCM, and other cancers. Conversely, low *ULK3* expression was associated with worse overall survival in 13 cancer types, including BLCA, HNSC, and STAD. These findings indicate that *ULK3* may exert differential effects across cancer types. Furthermore, based on survival data from the TCGA database, high *ULK3* expression was consistently associated with poor prognosis in ACC, KIRC, SKCM, LAML, and UVM. Low *ULK3* expression was also correlated with worse overall survival in BLCA, HNSC, KIRP, STAD, and other cancers. This seemingly paradoxical phenomenon may reflect the functional heterogeneity of ULK3 across different tumor backgrounds. It may also be related to differences in tumor stage, molecular subtype, and microenvironment composition [8]. For instance, in HNSC, loss of ULK3 may indirectly promote tumor adaptive survival by derepressing certain autophagic processes [12]. Therefore, the prognostic value of *ULK3* should be interpreted in a cancer type-specific context. Its bidirectional characteristics do not diminish its potential value as a biomarker. Instead, they highlight the need for cancer type-specific and individualized analyses in clinical applications [13].

Our analysis revealed a major genomic basis for *ULK3* upregulation. Copy number amplification was the most prevalent form of genetic alteration. As one of the most common genetic alterations in cancer genomes, copy number amplification can increase the copy number of specific genes. This process significantly affects cell growth regulation, repair mechanisms, and therapeutic responses [14]. Many studies have shown that copy number amplification is not a random event. It is closely associated with specific clinicopathological characteristics, treatment resistance, and patient prognosis [15]. A strong positive association between copy number gain and mRNA expression was observed in 18 cancers. This finding suggests that the gene dosage effect may be a major mechanism underlying *ULK3* upregulation [16]. In addition, missense mutations were the main mutation type. Among them, the A206V site located in the protein kinase (Pkinase) domain deserves special attention. ULK3 is a serine/threonine protein kinase. Its N-terminal Pkinase domain is the catalytic core responsible for ATP binding and substrate phosphorylation. ULK3 also plays important roles in autophagy, cell division, and Sonic Hedgehog (Shh) signal transduction [17,18]. This domain has a typical serine/threonine kinase fold. It contains the activation loop and key catalytic residues, such as Lys-43 [19]. However, these interpretations remain speculative. Direct functional evidence, such as kinase activity assays or phosphoproteomic profiling, is still lacking to confirm the biological impact of this mutation. The functional significance of the recurrent A206V missense mutation identified in our pan-cancer analysis also remains unclear. Although this mutation is located within the kinase domain and is predicted to affect kinase activity based on structural modeling, no experimental validation has yet been performed. It remains unknown whether A206V affects ULK3 enzymatic activity, substrate phosphorylation, or downstream signaling. Future studies using site-directed mutagenesis combined with kinase activity assays and functional readouts are warranted to clarify the biological consequences of this mutation [20]. Co-occurrence gene analysis suggested that *SCAMP2*, *CSK*, and other genes may be synergistically dysregulated with *ULK3* and participate in oncogenic pathways. This synergistic dysregulation may affect transcriptional activity, post-translational modification, or the interaction between ULK3 and its substrates through shared genetic regulatory elements, epigenetic modifications, and overlapping signaling pathways [21]. These findings provide molecular evidence for understanding the mechanism of ULK3 activation in cancer. They also lay a foundation for developing intervention strategies targeting ULK3 or its interaction network.

An in-depth analysis of genes co-expressed with *ULK3* in five representative cancers showed that these genes were highly enriched in RNA splicing and processing, methylation modification, mitochondrial energy metabolism, and oxidative phosphorylation pathways. This functional spectrum has important pathophysiological implications. First, abnormal RNA metabolism, especially dysregulated RNA splicing, has been recognized as an essential hallmark of tumorigenesis. *ULK3* co-expressed genes were enriched in the RNA splicing pathway. This finding suggests a potential association between *ULK3* and RNA splicing regulation, although functional experiments are still needed for validation. Notably, recent studies have characterized two major alternatively spliced isoforms of *ULK3*. The full-length transcript ENST00000440863.7 (*ULK3*-201) encodes a 472-amino-acid protein. The truncated transcript ENST00000569437.5 (*ULK3*-220) lacks two amino acids, valine and lysine (VK), at the C-terminus of the microtubule-interacting and transport (MIT) domain. This splicing switch is driven by the rs12898397 (T > C) genetic variant in exon 14. This variant weakens the canonical splice donor site and enhances a cryptic splice donor site, resulting in the skipping of six nucleotides [22]. AlphaFold 3.0 predictions indicate that loss of the VK dipeptide causes substantial structural divergence in the C-terminal region (RMSD = 5.81 Å). The MIT domain is critical for ULK3-mediated regulation of autophagy and Sonic Hedgehog signaling [16]. Therefore, splicing-induced structural alterations may affect the interaction of ULK3 with downstream effectors, such as SUFU and GLI proteins. These changes may further modulate its tumor-related functions. However, the functional consequences of these splicing events in cancer pathogenesis require further experimental investigation.

Numerous studies have shown that dysregulated RNA splicing can promote tumor cell growth, inhibit apoptosis, and enhance migration and metastasis. It can also affect immune surveillance by generating new antigens. Thus, RNA splicing disorder may drive the onset and progression of malignancies through multiple dimensions [23]. Second, *ULK3* expression was positively correlated with m1A, m5C, and m6A RNA-modifying enzymes. This finding suggests that *ULK3* may be involved in epitranscriptomic regulation. Moreover, the enrichment of oxidative phosphorylation and thermogenesis pathways, together with the correlation between *ULK3* and stemness indices, suggests that ULK3 may influence mitochondrial metabolic reprogramming and help maintain stemness characteristics [24].

Analysis of the tumor microenvironment showed that *ULK3* levels were significantly negatively correlated with Stromal Score, Immune Score, and ESTIMATE Score in a subset of cancers, including GBM, BRCA, LUAD, PRAD, LUSC, and BLCA. These findings suggest an immunologically “cold” phenotype in these cancer types. However, opposite correlations were observed in other tumor types, such as UVM and LAML. This result indicates that the immune associations of *ULK3* are largely cancer type-dependent. Such a microenvironment is often associated with immunotherapy resistance and poor prognosis [13]. *ULK3* was positively associated with the infiltration of M2-type tumor-associated macrophages in eight cancers. It was also negatively correlated with regulatory T cells in six cancers. M2 macrophages are important immunosuppressive cells that promote angiogenesis, tissue remodeling, and immune escape [25,26]. ULK3 may induce M2 polarization through the chemokine network, as reflected by its negative correlations with multiple chemokine receptors. This process may contribute to the establishment of an immunosuppressive barrier. However, functional experiments are still needed to validate its mechanistic role. In addition, *ULK3* was co-expressed with immune checkpoint molecules, such as *CD274* (PD-L1) and *CTLA4*, in 25 cancers. IFN-γ treatment also downregulated *ULK3* expression. These findings suggest that ULK3 may be involved in adaptive immune resistance. They further indicate that ULK3 may serve as a potential target for combination immunotherapy. However, co-targeting ULK3 requires further preclinical investigation.

Tumor mutational burden (TMB) and microsatellite instability (MSI) are key biomarkers for predicting the efficacy of immune checkpoint inhibitors [27]. Our study found that *ULK3* was positively correlated with TMB in KIRC and READ. *ULK3* was also positively correlated with MSI in LGG, LUAD, and UCEC. These correlations provide a theoretical rationale for considering *ULK3* as a candidate biomarker in immunotherapy. However, direct clinical evidence is currently lacking. It should be noted that the associations between *ULK3* expression and TMB/MSI are correlative. These associations do not directly demonstrate that *ULK3* can predict immunotherapy efficacy. Although TMB and MSI are established biomarkers for response to immune checkpoint inhibitors, the role of ULK3 in immunotherapy efficacy requires validation in patient cohorts receiving immunotherapy [27]. However, the association was cancer-specific, as shown by the negative association with MSI in DLBC. This finding again emphasizes the need for individualized analysis. Combined with the positive correlation between *ULK3* and multiple immune checkpoint blockade-related proteins, especially in UVM and DLBC, we hypothesize that high *ULK3* expression may not only contribute to an immunosuppressive microenvironment but also coexist with a high mutation burden. This pattern may form a paradoxical state of “immune exhaustion with potential responsiveness.” Therefore, *ULK3* may serve as a candidate biomarker with potential dual clinical implications. Its high expression is correlated with poor clinical outcomes and may also be associated with immunotherapy sensitivity. However, these hypotheses require validation in prospective clinical cohorts.

The analyses described above are entirely computational and represent the bioinformatics component of this study. To further explore the functional relevance of ULK3 in tumors under experimental conditions, we first performed subcellular localization assays. Cellular localization experiments showed that full-length ULK3 and its different domains were distributed in both the nucleus and cytoplasm. However, the N-terminus was mainly retained in the cytoplasm. This distribution pattern suggests that ULK3 may participate in diverse cellular processes. However, the specific functions of individual domains require further investigation. In prostate cancer PC-3 cells, *ULK3* overexpression significantly promoted cell migration and proliferation. These results directly supported its cancer-promoting function in this cellular model. Finally, CMap screening and molecular docking predicted Cephaeline as a potential ULK3-binding compound. This finding suggests possible ULK3-activating activity. Although this prediction is promising, it is based solely on computational simulations and requires pharmacological validation at both cellular and animal levels.

This study has several limitations. First, the public datasets used in this study were derived from multiple independent cohorts, which differed in sample size and algorithmic approach. To mitigate this limitation, we used multiple databases, including TCGA, GTEx, and CPTAC, for cross-validation according to different analytical purposes. Second, the correlation analyses involving immune infiltration, stemness indices, and therapeutic markers reflect only statistical associations. Therefore, all bioinformatics findings should be considered hypothesis-generating rather than conclusive. Third, Cephaeline was preliminarily identified as a potential ULK3-targeting compound through bioinformatics prediction and molecular docking. However, this finding is based solely on computational approaches. The regulatory effects of Cephaeline on ULK3 activity and the underlying mechanisms require further in vitro and in vivo validation. Fourth, the cellular functional experiments were confined to a single cell line, PC-3. This model may not fully reflect tumor heterogeneity or the tissue-specific roles of ULK3. Future studies should include multiple cell lines, animal models, and prospective clinical cohorts to further evaluate the clinical value of ULK3. Fifth, the immune-related correlations of *ULK3* were not consistent across cancer types. The observed correlations varied depending on the tumor context. For instance, a recent study reported that *ULK3* was significantly negatively correlated with macrophage infiltration in esophageal cancer [12]. In contrast, our analysis in prostate cancer revealed a positive correlation between *ULK3* and M2 macrophage infiltration. Therefore, our findings do not support a universal immunosuppressive role of ULK3. Future studies should investigate the mechanistic basis of this cancer type-specific heterogeneity. Sixth, our findings regarding the potential role of *ULK3* as a predictor of immunotherapy response are based solely on correlations with TMB, MSI, and immune checkpoint gene expression. These analyses did not include patient cohorts treated with immune checkpoint inhibitors. Therefore, the clinical value of *ULK3* as a predictive biomarker remains speculative. Prospective studies are required to validate its mechanism of action [12]. Seventh, although our genomic alteration analyses identified recurrent mutations and copy number variations of *ULK3*, the functional relevance of these alterations has not been experimentally validated. This limitation is particularly relevant to the A206V missense mutation. The predicted structural effects are based on computational modeling and require direct biochemical and cellular assays to confirm their biological significance.

In summary, our multidimensional pan-cancer analysis revealed that *ULK3* is overexpressed in most cancers and is driven in part by copy number amplification. *ULK3* expression is closely associated with poor prognosis, enhanced tumor stemness, immunosuppressive microenvironment formation, and abnormal RNA metabolism. *ULK3* may serve not only as a potential pan-cancer diagnostic and prognostic marker but also as a potential auxiliary indicator for predicting immunotherapy response. In prostate cancer, ULK3 also showed potential value as a therapeutic target. Future intervention strategies targeting ULK3, whether through small-molecule inhibitors or activators, should be analyzed according to the specific cancer context.

## 4. Materials and Methods

### 4.1. Analysis of ULK3 Gene and Protein Expression

mRNA expression data were retrieved from TCGA. All analyses were performed using R software (version 4.2.1). Differential expression was evaluated using the edgeR package based on raw RNA-seq counts. The data were transformed as log2(TPM + 1). Differential expression analysis was performed by comparing tumor tissues with matched adjacent normal tissues. Student’s *t*-test was used, and statistical significance was defined as *p* < 0.05. Box plots were generated in R using the ggplot2 package. *ULK3* mRNA expression differences between tumor samples and paired adjacent non-tumor tissues were also evaluated using the Gene_DE module of TIMER2.0 (Tumor Immune Estimation Resource 2.0) [28,29]. ULK3 protein expression levels in tumor tissues and normal tissues were evaluated using the UALCAN website [30]. Immunohistochemical images of eight tumor types and their corresponding normal tissues were obtained from The Human Protein Atlas (HPA) public database [31]. These images were used to assess differences in ULK3 protein expression. The images are presented as representative staining patterns. No quantitative statistical test was applied because raw intensity data were not available from the HPA platform.

### 4.2. Survival and Prognosis Analysis of ULK3

Using the Kaplan–Meier plotter database, we assessed the association between *ULK3* expression and patient survival across various cancer types. This study examined the relationship between *ULK3* expression levels and three key clinical endpoints: overall survival (OS), disease-specific survival (DSS), and progression-free interval (PFI). For each cancer type, patients were divided into *ULK3* high-expression and low-expression groups according to the median *ULK3* mRNA expression level. Kaplan–Meier survival curves were used to evaluate the association between *ULK3* expression and survival outcomes across different cancer types. A two-sided log-rank test was used for group comparisons. Univariate Cox proportional hazards regression was used to calculate hazard ratios (HRs) and 95% confidence intervals (CIs). To determine whether *ULK3* expression served as an independent prognostic factor, multivariate Cox regression analyses were also performed. *ULK3* expression and available clinicopathological covariates, including age, tumor stage, pathological grade, and sex, were incorporated into the models for each cancer type in the TCGA database. Statistical significance was defined as a two-sided *p*-value < 0.05.

### 4.3. Analysis of Genetic Alterations in ULK3

The cBioPortal online database was used to explore the genetic alteration characteristics of *ULK3* [32]. The “Pan-Cancer Studies” section was selected for query. The “Cancer Types Summary” module was used to obtain the overall alteration frequency of *ULK3* across cancer types. The “Mutations” module was used to obtain mutation types, mutation sites, and mutation counts. The “OncoPrint”, “Cancer Types Summary”, and “Mutations” modules were used to explore gene alterations and mutation locations [33]. The Gene_Mutation module of TIMER2.0 and SangerBox were used to determine the mutation types and mutation frequencies of *ULK3* across various tumors. The mRNA expression of *ULK3* and the corresponding copy number changes were analyzed using the SangerBox portal. The correlation between *ULK3* expression and somatic mutations was obtained from CAMOIP by selecting the “mutation landscape” section.

### 4.4. Clinical Relevance of ULK3 Alternative Splicing

To evaluate the clinical significance of alternative splicing (AS) events involving *ULK3*, we interrogated the ClinicalAS OncoSplicing module [34], which integrates data from the SplAdder and SpliceSeq projects. We employed PanPlot to visualize the percent-spliced-in (PSI) values of *ULK3* across tumor samples from The Cancer Genome Atlas (TCGA) and matched normal tissues from the Genotype-Tissue Expression (GTEx) project. PanDiff plots were used to compare PSI values between tumor tissues and their paired adjacent normal tissues—or GTEx-derived normal controls—for AS events recurrently detected in more than three distinct cancer types. Finally, Kaplan–Meier survival analysis was performed to assess the prognostic value of these pan-cancer *ULK3* splicing events.

### 4.5. Enrichment Analysis of ULK3 Co-Expressed Genes

Genes co-expressed with *ULK3* and their corresponding Pearson correlation coefficients were obtained from cBioPortal by selecting the “Co-expression” option. Genes with a Pearson correlation coefficient greater than 0.3 and a positive correlation with *ULK3* were selected for Gene Ontology (GO) term and Kyoto Encyclopedia of Genes and Genomes (KEGG) pathway enrichment analyses. These analyses were performed using the Database for Annotation, Visualization, and Integrated Discovery (DAVID). To further refine gene selection for heat map visualization, *ULK3*-positive co-expressed genes were ranked according to their Pearson correlation coefficients. The top 30 genes with the strongest positive correlations with *ULK3* across the five cancer types were selected. Each selected gene was required to show consistent positive correlation patterns in at least four of the five cancers to ensure cross-cancer reproducibility. The GO and KEGG gene clustering results were obtained from the corresponding websites. Venn diagrams, GO chord diagrams, and GO/KEGG pathway plots were generated using SRplot (Science and Research Online Plot), a user-friendly online platform that integrates more than 100 commonly used data visualization and plotting functions. The Gene Expression Profiling Interactive Analysis 2 (GEPIA2) database was used to visualize the heat map of *ULK3*-positive co-expressed genes by selecting the “Multiple Gene Comparison” section.

### 4.6. Correlation Analysis of ULK3 Stemness Indices

This study assessed the relationships between *ULK3* expression and multiple stemness-related indices, including DNAss, EREG-METHss, DMPss, ENHss, RNAss, and EREG-EXPss. Pearson correlation coefficients (Pearson R) between *ULK3* and each stemness-related index were obtained from the SangerBox online platform. MicroBioinfo was used to generate lollipop plots for visualization. In addition, SangerBox was used to perform Pearson correlation analysis between *ULK3* expression and various RNA modification regulators. A corresponding heat map was generated to visualize these correlations.

### 4.7. Correlation Analysis of Immune Scores and Immune Cell Infiltration

The correlations between *ULK3* expression and Stromal Score, Immune Score, and ESTIMATE Score were assessed using the SangerBox online platform. The CIBERSORT algorithm was used to examine the associations between *ULK3* expression and different immune cell types, including B cells, CD4+ T cells, CD8+ T cells, myeloid dendritic cells (MDCs), macrophages, and neutrophils (NE). TIMER2.0 was used to generate scatter plots showing the correlations between *ULK3* expression and immune cell infiltration. These analyses included both tumor purity and purity-adjusted correlations of ULK3 with B cells, CD4+ T cells, CD8+ T cells, MDCs, macrophages, and NE in five cancer types, including BRCA, LIHC, LUSC, SARC, and TGCT.

### 4.8. Investigation of the Immune Effects of ULK3 in the Pan-Cancer Microenvironment

We integrated publicly available datasets from the previous literature to explore the relationship between *ULK3* expression and immune checkpoint molecule expression [35]. Spearman correlation coefficients were calculated between ULK3 and immune checkpoint genes, including *CD274*, *CTLA4*, *LAG3*, *PDCD1*, *TIGIT*, *HAVCR2*, and other related genes. Statistical significance was defined as *p* < 0.05. In the immune subtype module of the tumor immune microenvironment database, we analyzed the relationship between *ULK3* expression and immune subtypes. We also evaluated the relative expression levels of *ULK3* among these immune subtypes. Heat maps were generated to examine the correlations between *ULK3* expression and diverse immunomodulatory molecules, including chemokines, their corresponding receptors, and immunostimulatory factors within predefined immunomodulator and chemokine gene sets. To assess the effect of cytokine treatment on *ULK3* expression, we used the Tumor Immune Syngeneic Mouse (TISMO) online tool [35]. This tool was used to compare *ULK3* gene expression levels in cell lines before and after cytokine treatment.

### 4.9. Correlation Analysis of ULK3 with Immunotherapy Biomarkers and Treatment Response

The SangerBox online platform was used to calculate Pearson correlation coefficients between *ULK3* expression and tumor mutational burden (TMB), microsatellite instability (MSI), and predicted neoantigen (NEO) load. Radar plots were generated using Microsoft Excel. In addition, the correlations between *ULK3* expression and immunomodulatory genes, including immunoinhibitory and immunostimulatory genes, were evaluated across different tumors in the TCGA cohort using the SangerBox online platform. Furthermore, the relationships between *ULK3* expression and chemokines or major histocompatibility complex (MHC) molecules were assessed using Tumor–Immune System Interactions and DrugBank (TISIDB). TISIDB is a platform that integrates multiple tumor immunology data resources. The “chemokines” and “immunomodulators” sections were selected for analysis. *ULK3* expression levels in patients who responded to immunotherapy and in those who did not respond were also obtained from TISIDB by selecting the “immunotherapy” category.

### 4.10. Cell Culture

Human embryonic kidney HEK 293T cells, human prostate cancer PC-3 cells, DMEM medium, Ham’s F-12 medium, gentamicin, and 0.25% trypsin were obtained from Servicebio Biotechnology Co., Ltd. (Wuhan, China). Fetal bovine serum was obtained from Roya Biotechnology Co., Ltd. (Lanzhou, China).

### 4.11. Plasmid Construction

The *ULK3* coding sequence (CDS) was retrieved from NCBI. According to the restriction sites of the pEGFP-C2 empty vector sequence and the characteristics of the *ULK3* CDS, XhoI and BamHI were selected as the upstream and downstream restriction sites, respectively. The primer sequences used for amplifying the full-length *ULK3* and its various truncated forms are shown in Table 1.

The PCR reaction system was prepared according to the instructions for DNA polymerase 2 × Flash PCR Master Mix (Dye) (Cowin Biotech Co., Ltd., Jiangsu, China). PCR amplification was performed using a PCR instrument (Bio-Rad Laboratories, California, USA). The PCR program was set as follows: denaturation at 98 °C for 10 s, annealing at 61 °C for 5 s, and extension at 72 °C for 50 s. The samples were then maintained at 4 °C. The amplified products were separated by electrophoresis on a 1% agarose gel. Bands of the expected size were excised and purified to obtain the target DNA fragments.

For restriction enzyme digestion, the pEGFP-C2 (Laboratory storage) empty vector was digested with XhoI (TransGen Biotech, Beijing, China) and BamHI (TransGen Biotech, Beijing, China) at 37 °C for 30 min. The linearized vector was separated by electrophoresis on a 1% agarose gel and recovered after gel excision.

For ligation, the purified DNA fragments and the linearized vector were ligated using homologous recombinase. The ligation system was prepared according to the manufacturer’s instructions for homologous recombinase. The reaction was performed at 37 °C for 30 min and then immediately placed on ice.

For transformation and identification, competent cells (TransGen Biotech, Beijing, China) were thawed on ice. The homologous recombination product was gently added to the competent cells. The tube wall was tapped gently to mix the contents, and the reaction was kept on ice for 30 min. Heat shock was performed at 42 °C for 45 s, followed by immediate cooling on ice for 3 min. Then, 900 μL of liquid LB medium was added, and the cells were shaken at 37 °C for 1 h. After centrifugation at 5000× *g* for 5 min, the supernatant was removed. The bacterial pellet was resuspended in fresh LB medium and evenly plated onto kanamycin-resistant solid LB agar plates. The bacterial culture was incubated at 37 °C for 12–16 h. Monoclonal colonies were selected and cultured with shaking. Plasmids were extracted and identified by double-enzyme digestion.

### 4.12. Cell Slide Staining and Laser Confocal Imaging

The pEGFP-C2 empty vector, *ULK3*-FL plasmid, and each truncated *ULK3* plasmid were transfected into 293T cells using Lipofectamine 3000 (Invitrogen, Carlsbad, CA, USA). Because the plasmids carried the EGFP fluorescent tag, fluorescence was observed 24 h after transfection to determine transfection efficiency. Subsequently, the cells were permeabilized with 0.1% Triton X-100 (LABGIC, Hefei, China) for 15 min at room temperature. The nuclei were stained with 100 ng/mL DAPI (Fude Biotechnology Co., Ltd., Hangzhou, China) for 15 min. The cells were then washed three times with PBS (Servicebio Biotechnology Co., Ltd., Wuhan, China). The slides were mounted without an antifade reagent. After air drying, the slides were imaged using laser confocal microscopy (Nikon Corporation, Tokyo, Japan).

### 4.13. Scratch Test

PC-3 cells in the logarithmic growth phase were cultured in 35-mm cell culture dishes. When the cells reached approximately 70% confluence, they were transfected with the pEGFP-C2 empty vector or the *ULK3*-FL plasmid. At 24 h after transfection, fluorescence was observed to verify transfection efficiency. Scratch wounds were then created in the cell monolayer using a 200 μL pipette tip. Cells were photographed under a microscope at 0 h and 36 h after scratching. ImageJ (version 1.5.1) was used to measure the migration distance. The wound closure rate was calculated for both the empty vector control group and the *ULK3*-FL experimental group.

### 4.14. MTT Assay

PC-3 cells in the logarithmic growth phase were seeded into 96-well plates at a density of 5000 cells per well. The cells were then transfected with either the pEGFP-C2 empty vector or the plasmid expressing full-length *ULK3*. Fluorescence was observed 24 h after transfection to confirm successful transfection. At 48 h after transfection, MTT reagent (LABGIC, Hefei, China) was added. After incubation at 37 °C for 4 h, DMSO (Sigma-Aldrich, Darmstadt, Germany) was added, and the plate was incubated for 30 min. Absorbance at 490 nm was measured using a microplate reader (BioTek, Vermont, USA).

### 4.15. Screening of ULK3-Activating Drugs

The “Query” tool in CMap was used to identify potential compounds that stimulate ULK3. The analysis was based on the top 100 most significantly altered genes, including both upregulated and downregulated genes, stratified according to the median expression level of *ULK3*. After data collection, a heat map was generated for the top 30 compounds. Scatter plots were created using Microsoft Excel to evaluate the potential biological mechanisms of compound activity. Subsequently, *ULK3* expression levels and GI50 values, which represent the concentration required to inhibit cell proliferation by 50%, were retrieved from the CellMiner database. Data processing was performed using R with the readxl, impute, and lima packages. Plots were generated using the MicroBioinfo online platform. The three-dimensional structures of ULK3 and the corresponding compounds were obtained from the PDB and PubChem databases, respectively. Docking studies were conducted using AutoDock version 4.2.6. The results were visualized using PyMOL version 3.1.0.

### 4.16. Statistical Analysis

Statistical analyses were performed using R software version 4.2.1. The unpaired Wilcoxon rank-sum test or Welch’s *t*-test was used for comparisons between two groups. The Kruskal–Wallis test was used for comparisons among multiple groups. Pearson or Spearman correlation analysis was performed using the corr. test function, with the method set as “pearson” or “spearman”, respectively. Survival analysis was performed using the Kaplan–Meier method and the log-rank test. The R packages survival version 3.3.1 and survminer version 0.4.9 were used for survival analysis. Analyses involving multiple comparisons, including differential expression and correlation analyses, and survival analyses for each cancer endpoint, including OS, DSS, and PFI, were adjusted using the Benjamini–Hochberg false discovery rate (FDR) method. A *q*-value < 0.05 was considered statistically significant. Asterisks in the figures indicate statistical significance as follows: * *p* < 0.05, ** *p* < 0.01, and *** *p* < 0.001. For multi-platform data integration, we used a strategy combining independent analysis with cross-validation. The same analytical content was processed independently across different platforms, such as TCGA plus GTEx, TIMER2.0, and CPTAC. Each platform used its own standardized algorithm, and the results were presented in independent charts. Data from each platform had been normalized and preprocessed by the corresponding database. Therefore, this study did not perform secondary cross-platform data mixing. Key conclusions were cross-validated by assessing the consistency of results across multiple platforms to reduce the influence of platform-specific bias. Please refer to Supplementary Table S1 for detailed basic information on the public datasets utilized in this research.

## 5. Conclusions

In summary, *ULK3* is upregulated in a range of cancers and is closely associated with poor prognosis, genomic variation, immunosuppressive microenvironment formation, and cancer-promoting functions. ULK3 may serve as a potential prognostic biomarker and therapeutic intervention point, especially in prostate cancer. However, the molecular mechanisms underlying the opposite prognostic trends of *ULK3* across different cancer types remain unclear. In some cancers, low *ULK3* expression is also associated with poor outcomes. Direct evidence that ULK3 drives tumor progression through RNA modification, metabolic reprogramming, or immune checkpoint regulation remains to be established through combined in vitro and in vivo functional experiments and clinical cohort studies. In addition, the anticancer effect of Cephaeline-mediated ULK3 activation and the underlying pathways in prostate cancer require further validation.

## Figures and Tables

**Table 1:** Primer sequences.

Primer Name	Primer Sequence
*ULK3*-FL-F	AAGTCCGGCCGGACTCAGATCTCGAGTATGGCGGGGCCC
*ULK3*-FL-R	TTATCTAGATCCGGTGGATCCTCACTGAAGGGTGC
*ULK3*-N-R	TTATCTAGATCCGGTGGATCCTCAGATGAGGTAGATATTG
*ULK3*-N + MIT2-R	TTATCTAGATCCGGTGGATCCTCAATGAAGACACGCGCCACCT
*ULK3*-MIT-F	AAGTCCGGCCGGACTCAGATCTCGAGACAGCAATTAGCTAG
*ULK3*-C-F	AAGTCCGGCCGGACTCAGATCTCGAGATGGAGTTTTGCGC

## Data Availability

The original contributions presented in the study are included in the article/Supplementary Materials. Further inquiries can be directed to the corresponding author.

## Supplementary Materials

The following supporting information can be downloaded at https://www.mdpi.com/article/10.3390/ijms27136040/s1. References [28,29,30,32,36,37,38,39,40,41,42,43,44] are cited in the supplementary materials.
