# Supplementary material for: The Role of ULK3 in Cancer Progression: A Pan-Cancer Bioinformatics Analysis Integrated with Experimental Validation in Prostate Cancer
Source: Int J Mol Sci. 2026 Jul 5;27(13):6040. doi: 10.3390/ijms27136040 (PMC13361878; doi:10.3390/ijms27136040)
Supplement: Supplementary file 1 [file ijms-27-06040-s001.zip › Supplementary Figures.pdf]

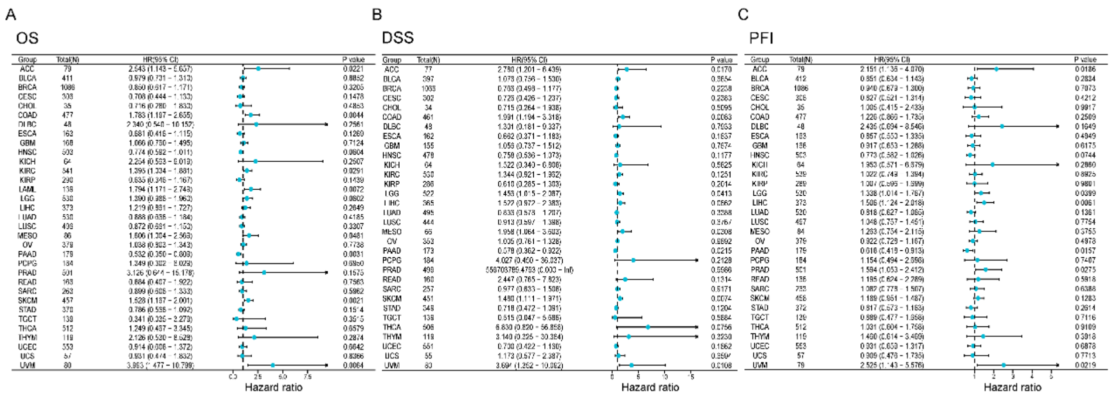

Supplementary Figure S1 Univariate Cox regression analyses ULK3 expression in pan-cancer. (A) The relationship between ULK3 genes and overall survival time in days in TCGA tumor and nontumor. (B) The relationship between ULK3 gene and disease-specific survival in TCGA tumor and nontumor. (C) The relationship between ULK3 gene and prognosis-free interval in TCGA tumor and nontumor. Hazard ratio (HR) value > 1 represents risk factor, whereas HR value < 1 represents favorable factor.

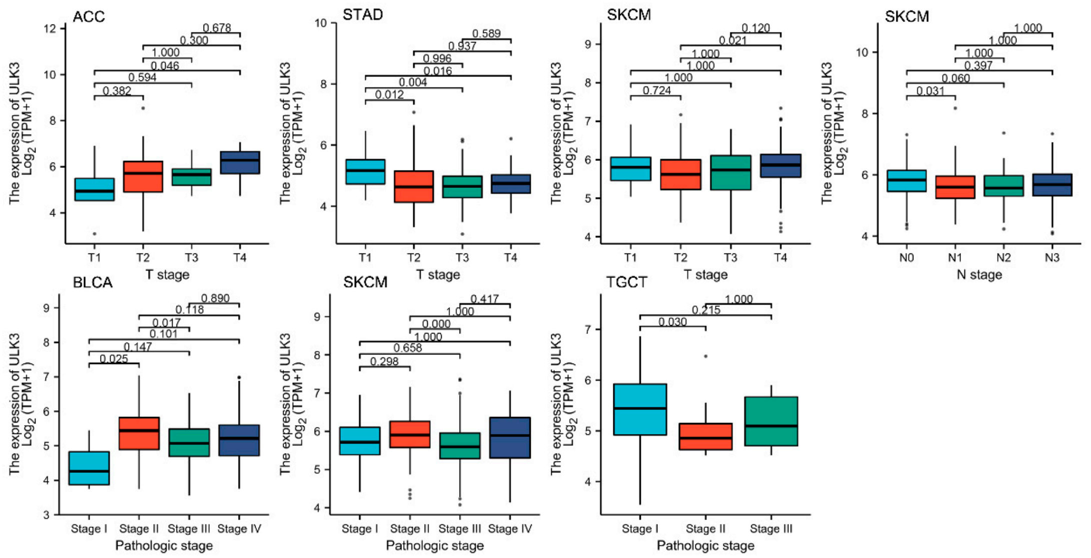

Supplementary Figure S2 The correlation between the mRNA expression level of ULK3 and patients' clinicopathological features progression in pan-cancer.

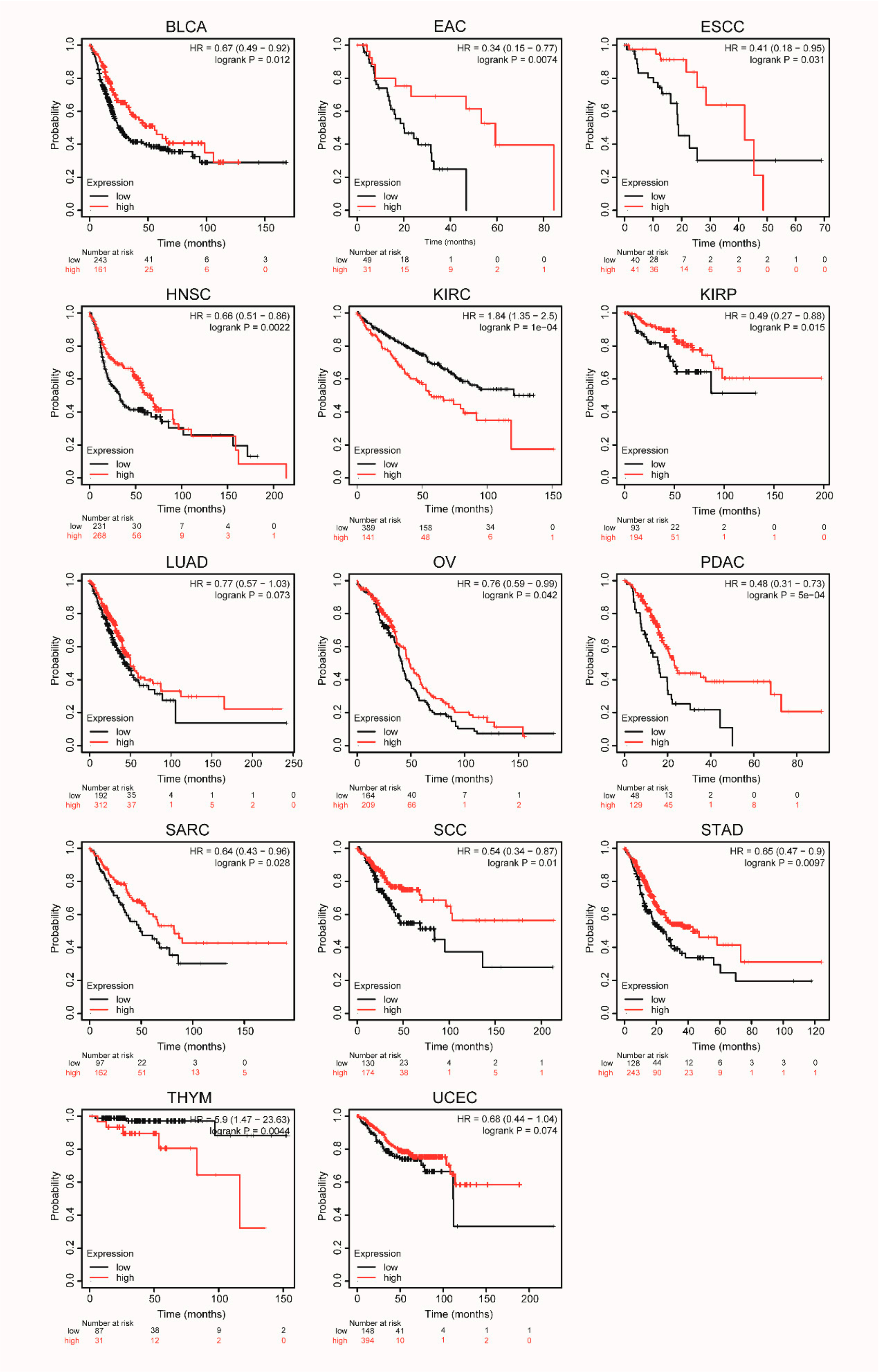

Supplementary Figure 3 Kaplan–Meier analysis of the association between ULK3 expression and OS.
