# Supplementary material for: The Role of ULK3 in Cancer Progression: A Pan-Cancer Bioinformatics Analysis Integrated with Experimental Validation in Prostate Cancer
Source: Int J Mol Sci. 2026 Jul 5;27(13):6040. doi: 10.3390/ijms27136040 (PMC13361878; doi:10.3390/ijms27136040)
Supplement: Supplementary file 1 [file ijms-27-06040-s001.zip › Supplementary Tables.pdf]

Supplementary Table S1 Summary of basic information on public datasets used in this study

Data set	Sample size	Tumor type	Normal control	Data processing method	References
TCGA	Total number of TCGA samples	33 types of TCGA pan-cancer	TCGA paired normal tissues of each cancer type	Log2(TPM + 1) transformation; edgeR differential analysis; Cox regression + log-rank survival analysis	[1]
GTEx	17382	No (only normal tissues)	31 types of normal tissues	Normalized TPM; merged with TCGA data as normal controls	[2]
CPTAC	Number of paired cases for each cancer type	BRCA、COAD、GBM、HNSC、LIHC、LUAD、LUSC、UCEC	TCGA paired normal tissues for each cancer type	Proteomics data; log2 normalization; calculation of Z value; Welch's t-test	[30]
TIMER 2.0	Total number of TCGA samples	33 types of TCGA pan-cancer	TCGA paired normal tissues of each cancer type	TPM normalized (without log transformation); CIBERSORT assessment of 22 immune cells; Spearman correlation; FDR correction	[28][29][3]
cBioPortal	Total number of TCGA samples	33 types of TCGA pan-cancer	Not available (only tumor genomic data)	Mutation frequency, mutation type (missense/missense/splice site), copy number variation (processed by GISTIC), structural variation; co-expression analysis	[32]
UALCAN	Total number of TCGA samples	33 types of TCGA pan-cancer	TCGA paired normal tissues of each cancer type	TCGA-assembler obtains DNA methylation data; calculates average β value in the promoter region (1500bp upstream to 200bp downstream of TSS); performs Welch's t-test	[30]
Kaplan-Meier Plotter	Total TCGA samples + GTEx cohort	33 types of TCGA pan-cancer	Not applicable (only tumor clinical survival cohort)	Cox regression; log-rank test; median grouping method	[4]
SangerBo	Total	33 types of	TCGA paired	Multimodal integration;	[5]

x	TCGA samples + GTEx cohort	TCGA pan-cancer	normal tissues of each cancer type + GTEx multi-tissue normal samples	ESTIMATE calculates immune/matrix scores; maftools calculate TMB/MSI/NEO; Pearson correlation	
GEPIA2	Total number of TCGA samples	33 types of TCGA pan-cancer	TCGA paired normal tissues of each cancer type	Log2(TPM + 1) transformation; Visualization of co-expressed gene heatmap	[6]
TISMO	Various human tumor cell lines	Extracellular cancer cell lines	No normal human tissue control	Comparison of ULK3 expression before and after cytokine (IFN-β, IFN-γ, TNF-α, etc.) treatment	[7]
CAMOIP	otal sample number of TCGA + ICI immunotherapy cohort	33 types of TCGA pan-cancer samples + multiple ICI immunotherapy clinical tumor cohorts;	TCGA paired normal tissues of each cancer type (the ICI cohort has no normal control for pairing)	Fisher's exact test for comparing mutation frequencies; TMB comparison	[8]
CellMiner	NCI-60	60 types of cancer cell lines	No normal human tissue control	Acquisition of drug GI50 values; processed using R language (readxl, impute, limma packages)	[9]

1. The Cancer Genome Atlas Research Network; Weinstein, J.N.; Collisson, E.A.; Mills, G.B.; Shaw, K.R.M.; Ozenberger, B.A.; Ellrott, K.; Shmulevich, I.; Sander, C.; Stuart, J.M. The Cancer Genome Atlas Pan-Cancer Analysis Project. *Nat Genet* **2013**, *45*, 1113–1120, doi:10.1038/ng.2764.
2. Carithers, L.J.; Moore, H.M. The Genotype-Tissue Expression (GTEx) Project. *Biopreservation and Biobanking* **2015**, *13*, 307–308, doi: 10.1089/bio.2015.29031.hmm.
3. Li, T.; Fan, J.; Wang, B.; Traugh, N.; Chen, Q.; Liu, J.S.; Li, B.; Liu, X.S. TIMER: A Web Server for Comprehensive Analysis of Tumor-Infiltrating Immune Cells. *Cancer Research* **2017**, *77*, e108–e110, doi: 10.1158/0008-5472.CAN-17-0307.
4. Lánczky, A.; Győrffy, B. Web-Based Survival Analysis Tool Tailored for Medical Research (KMplot): Development and Implementation. *J Med Internet Res* **2021**, *23*, e27633, doi:10.2196/27633.
5. Chen, D.; Xu, L.; Xing, H.; Shen, W.; Song, Z.; Li, H.; Zhu, X.; Li, X.; Wu, L.; Jiao, H.; et al. Sangerbox 2: Enhanced Functionalities and Update for a Comprehensive Clinical Bioinformatics Data Analysis Platform. *iMeta* **2024**, *3*, e238, doi:10.1002/imt2.238.
6. Tang, Z.; Kang, B.; Li, C.; Chen, T.; Zhang, Z. GEPIA2: An Enhanced Web Server for Large-Scale Expression Profiling and Interactive Analysis. *Nucleic Acids Research* **2019**, *47*, W556–W560, doi:10.1093/nar/gkz430.
7. Zeng, Z.; Wong, C.J.; Yang, L.; Ouardaoui, N.; Li, D.; Zhang, W.; Gu, S.; Zhang, Y.; Liu, Y.;

Wang, X.; et al. TISMO: Syngeneic Mouse Tumor Database to Model Tumor Immunity and Immunotherapy Response. *Nucleic Acids Research* **2022**, *50*, D1391–D1397, doi:10.1093/nar/gkab804.

8. Lin, A.; Qi, C.; Wei, T.; Li, M.; Cheng, Q.; Liu, Z.; Luo, P.; Zhang, J. CAMOIP: A Web Server for Comprehensive Analysis on Multi-Omics of Immunotherapy in Pan-Cancer. *Briefings in Bioinformatics* **2022**, *23*, bbac129, doi:10.1093/bib/bbac129.

9. Shank avaram, U.T.; Varma, S.; Kane, D.; Sunshine, M.; Chary, K.K.; Reinhold, W.C.; Pommier, Y.; Weinstein, J.N. CellMiner: A Relational Database and Query Tool for the NCI-60 Cancer Cell Lines. *BMC Genomics* **2009**, *10*, 277, doi:10.1186/1471-2164-10-277.

Supplementary Table S2 Analysis of transcriptional expression differences of ULK3 mRNA sample size

Cancer type	Tumor sample size	Normal sample size
ACC	79	0
BLCA	406	19
BRCA	1069	111
CESC	304	3
CHOL	35	9
COAD	454	41
DLBC	48	0
ESCA	162	11
GBM	160	0
HNSC	502	44
KICH	65	25
KIRC	72	532
KIRP	290	32
LAML	150	0
LGG	513	0
LIHC	371	50
LUAD	516	59
LUSC	501	49
MESO	87	0
OV	378	0
PAAD	178	4
PCPG	179	3
PRAD	497	52
READ	165	10
SARC	259	2
SKCM	469	1
STAD	375	32
TGCT	134	0
THCA	504	59
THYM	120	2
UCEC	545	23

UCS	57	0
UVM	80	0

Supplementary Table S3 Analysis of protein expression differences of ULK3 sample size

Cancer type	Tumor sample size	Normal sample size
BRCA	125	18
COAD	97	100
GMB	99	10
HNSC	108	71
LIHC	110	107
LUAD	165	165
LUSC	111	111
UCEC	100	31

Supplementary Table S4 ULK3 survival expression differential analysis sample size

Cancer type	Tumor sample size	Normal sample size
ACC	79	0
BLCA	406	19
BRCA	1069	111
CESC	304	3
CHOL	35	9
COAD	454	41
DLBC	48	0
ESCA	162	11
GBM	160	0
HNSC	502	44
KICH	65	25
KIRC	72	532
KIRP	290	32
LAML	150	0
LGG	513	0
LIHC	371	50
LUAD	516	59
LUSC	501	49
MESO	87	0
OV	378	0
PAAD	178	4
PCPG	179	3
PRAD	497	52
READ	165	10
SARC	259	2
SKCM	469	1

STAD	375	32
TGCT	134	0
THCA	504	59
THYM	120	2
UCEC	545	23
UCS	57	0
UVM	80	0

Supplementary Table S5 Genomic feature analysis sample size of ULK3

Cancer type	Sample size	Neutral	Loss	Gain
ACC	/	71	/	4
BLCA	407	381	8	16
BRCA	980	1004	29	50
CESC	/	269	/	21
COAD	282	278	4	4
COADREAD	372	368	5	4
ESCA	180	162	5	13
GBMLGG	/	646	6	5
HNSC	/	489	10	13
KIRC	334	/	/	/
KIRP	279	/	/	/
KIPAN	679	/	/	/
LGG	/	499	6	3
LIHC	356	349	7	11
LUAD	508	484	8	19
LUSC	485	459	10	28
MESO	/	82	/	5
OV	/	335	32	48
PAAD	168	169	4	4
PRAD	492	480	9	3
READ	90	/	/	/
SARC	234	228	15	14
SKCM	/	99	/	3
STAD	409	384	11	16
STES	589	546	16	29
THCA	487	/	/	/
UCEC	175	167	6	7
UCS	57	46	4	6

Supplementary Table S6 Sample size of ULK3 spliceosome in pan-cancer analysis

Cancer type	Tumor sample size	Normal sample size
ACC	72	0
BLCA	381	18
BRCA	1048	109

CESC	266	2
CHOL	35	9
COAD	279	40
DLBC	41	0
ESCA	147	11
GBM	167	0
HNSC	476	42
KICH	64	24
KIRC	381	72
KIRP	267	32
LGG	520	0
LIHC	324	46
LUAD	516	59
LUSC	499	47
MESO	79	0
OV	283	0
PAAD	174	4
PCPG	161	3
PRAD	472	51
READ	93	10
SARC	236	1
SKCM	466	1
STAD	306	32
TGCT	142	0
THCA	471	56
THYM	94	2
UCEC	146	21
UCS	48	0
UVM	80	0

Supplementary Table S7 Sample size for analysis of the association between ULK3 and cancer stem cell properties and RNA modificatio

Cancer type	Sample size
ACC	76
BLCA	403
BRCA	774
CESC	301
CHOL	36
COAD	271
COADREAD	358
DLBC	47
ESCA	179
GBM	51

GBMLGG	558
HNSC	512
KICH	65
KIRC	309
KIRP	268
KIPAN	642
LAML	170
LGG	507
LIHC	366
LUAD	451
LUSC	361
MESO	87
OV	9
PAAD	156
PCPG	176
PRAD	491
READ	87
SARC	253
SKCM	102
STAD	369
STES	548
TGCT	147
THCA	499
THYM	119
UCEC	173
UCS	57
UVM	79

Supplementary Table S8 Sample sizes for the association analysis between ULK3 and immune cell infiltration

Cancer type	Sample size
ACC	77
ALL	86
BLCA	405
BRCA	1077
CESC	291
CHOL	36
COAD	282
COADREAD	373
DLBC	46
ESCA	181
GBM	152
GBMLGG	656
HNSC	517

KICH	65
KIRC	528
KIRP	285
KIPAN	878
LAML	214
LGG	504
LIHC	363
LUAD	500
LUSC	491
MESO	85
OV	416
PAAD	177
PCPG	177
PRAD	495
READ	91
SARC	258
SKCM	452
STAD	388
STES	569
TGCT	132
THCA	503
THYM	118
UCEC	178
UCS	56
UVM	79

Supplementary Table S9 Sample size in the correlation analysis between ULK3 and immune checkpoint blocking proteins

Cancer type	Sample size
ACC	77
BLCA	407
BRCA	981
CESC	286
CHOL	36
COAD	282
COADREAD	372
DLBC	37
ESCA	180
GBM	149
GBMLGG	650
HNSC	498
KICH	66
KIRC	334
KIRP	279

KIPAN	679
LAML	126
LGG	501
LIHC	357
LUAD	509
LUSC	486
MESO	82
OV	303
PAAD	171
PCPG	177
PRAD	492
READ	90
SARC	234
SKCM	102
STAD	409
STES	589
TGCT	143
THCA	489
THYM	118
UCEC	175
UCS	57
UVM	79
